# Integrated Proteomic and Metabolomic Analyses Provide Insights Into Acquisition of Embryogenic Ability in *Agapanthus praecox*

**DOI:** 10.3389/fpls.2022.858065

**Published:** 2022-05-18

**Authors:** Jianhua Yue, Yan Dong, Songhu Liu, Yanan Jia, Chaoxin Li, Zhiyong Wang, Shoufu Gong

**Affiliations:** ^1^School of Horticulture, Xinyang Agriculture and Forestry University, Xinyang, China; ^2^School of Forestry, Xinyang Agriculture and Forestry University, Xinyang, China; ^3^College of Plant Science, Tarim University, Alar, China

**Keywords:** *Agapanthus praecox*, somatic embryogenesis, organogenesis, embryogenic callus, cell totipotency, cell pluripotency, cell differentiation

## Abstract

Somatic embryogenesis (SE) is an ideal model for plant cell totipotency. Transition from somatic cells to embryogenic cells is the key to SE. The poor frequency of embryogenic callus (EC) induction has limited the application of SE in many plants, such as *Agapanthus praecox*. We performed large-scale, quantitative proteomic and metabolomic analyses with different callus differentiation directions (SE and organogenesis) and stages (initial SE and repetitive SE) to better understand the morphological, physiological, and molecular characteristics of the acquisition of embryogenic ability in *A*. *praecox*. Integrated proteomic and metabolomic analyses suggested that callus differentiation direction was potentially regulated by pathways related to carbohydrate and energy metabolism (fatty acid metabolism, pyruvate metabolism, glycolysis/gluconeogenesis, pentose and glucuronate interconversions, starch and sucrose metabolism, galactose metabolism, carbon fixation pathways in prokaryotes, carbohydrate digestion and absorption, and fructose and mannose metabolism), chromatin accessibility and DNA methylation, reactive oxygen species responses and resistance (ascorbate and aldarate metabolism), and plant hormonal signaling. As a validation, we found that carbon source combination and plant hormone regulation in the culture medium significantly affected the acquisition of embryogenic ability, thereby inducing EC. Interestingly, plant hormonal signaling-related genes showed different expression patterns significantly when callus cultured with different carbon sources. Thus, our results suggested that energy supply and hormone signal transduction seemed to cooperatively contribute to the activation of embryogenic ability. Altogether, this study revealed valuable information regarding the molecular and biochemical changes that occurred during EC induction and provided valuable foundation for comprehensive understanding of the mechanisms associated with SE and organogenesis in *A*. *praecox*.

## Introduction

Plants display a remarkable capacity for somatic cell totipotency, as demonstrated by a single somatic cell prerequisite for reprogramming, and then develop into complete plants (Guo et al., 2019; Wang et al., 2020). Somatic embryogenesis (SE) is a cell differentiation process involved in dedifferentiation and redifferentiation through the reconstruction of somatic cells to generate somatic embryos (SEs; Yang and Zhang, 2010). SE is an important model for studying the totipotency of somatic cells in plants (Fehér, 2015; Su et al., 2021). Based on cellular totipotency, the embryogenic callus (EC) showed great superiority for micropropagation, cryopreservation, genetic transformation, embryological research in plants (Zhou et al., 2016; Wang et al., 2020). Much progress has been made in the elucidation of the underlying mechanisms of SE (Fehér, 2015; Horstman et al., 2017; Su et al., 2021). However, EC induction and plant regeneration are affected by many factors, including genotypes, explants, hormones, and concentrations of various substances in the induction medium (Guo et al., 2020; Wang et al., 2020, 2022). So far, the acquisition of EC has been shown as the critical process in plant SE (Wang et al., 2022). However, it is difficult to identify the cells capable of embryogenesis with morphological data. During the somatic to embryogenic transition, the cells must dedifferentiate, activate their cell division cycle, and reorganize their metabolic and physiological states (Kumaravel et al., 2017; Wang et al., 2020). Cell dedifferentiation results in the totipotency fate determination of somatic plant cells and embryogenesis initiation (Guo et al., 2020). The transition phase toward competent and embryogenic cell types is much less defined so far. Therefore, it is critical to track and dissect the specific cellular events associated with the acquisition of embryogenic competence in such highly refined systems (Guo et al., 2019).

Thus, detailed characterization of the embryogenic state, especially at the molecular level, is required to complement macromorphological and cytological observations of proliferating structures (EC and non-EC) generated following SE induction (Rutledge et al., 2017). Most previous studies revealed the differences between EC and non-EC and screened the developmental differences between zygotic embryogenesis and SE (Kumaravel et al., 2017; Aguilar-Hernández and Loyola-Vargas, 2018; Liu et al., 2019; Juarez-Escobar et al., 2021). Putative marker genes and transcription factors of the embryogenic state have been highlighted, including the ones involved in embryogenic induction in higher plants, particularly totipotency and commitment to SE, such as *somatic embryogenesis receptor kinases* (*SERKs*), *WUSCHEL* (*WUS*), *Agamous-like15* (*AGL15*), *Leafy cotyledon1* (*LEC1*), *LEC2, Baby Boom* (*BBM*), and *Fusca3* (*FUS3*; Yang et al., 2014; Wójcikowska and Gaj, 2015; Horstman et al., 2017; Orłowska et al., 2017; Wang et al., 2020; Su et al., 2021).

The development and application of omics technology, including transcriptomics, proteomics, and metabolomics, have significantly promoted the elucidation of mechanisms concerning plant SE in recent years (Marsoni et al., 2008; Yin et al., 2008; Kumaravel et al., 2017; Sun et al., 2018; Liu et al., 2019). However, both commonalities and personalities were identified within plants (Aguilar-Hernández and Loyola-Vargas, 2018; Tang et al., 2020; Wójcikowska et al., 2020). It was found that SE relative differentially expressed genes (DEGs) were mainly enriched in hormone synthesis and signal-related pathways in *Catalpa bungei* and *Gossypium hirsutum* (Sun et al., 2018; Liu et al., 2019). The interaction between hormones and stress signal-related differentially accumulated proteins (DAPs) was indispensable in the embryogenic fate determination in *Musa nana* (Kumaravel et al., 2017), while DAPs related to SE were mainly enriched in cell differentiation, stress, and glucose metabolism in *Zea mays* (Sun et al., 2013). Besides, proteins related to cellular metabolism, carbohydrate metabolism, oxidative stress response, glycolysis, and hormones were differentially accumulated during callus (CA) differentiation in *Oryza sativa* and *Vitis vinifera* (Marsoni et al., 2008; Yin et al., 2008). Moreover, a study combining high-throughput proteomics and metabolomics revealed the dynamic and complex network of EC formation in plants, which showed strong evidence of the capacity for embryogenic ability acquisition (Ge et al., 2017; Su et al., 2021).

**Figure 1:**
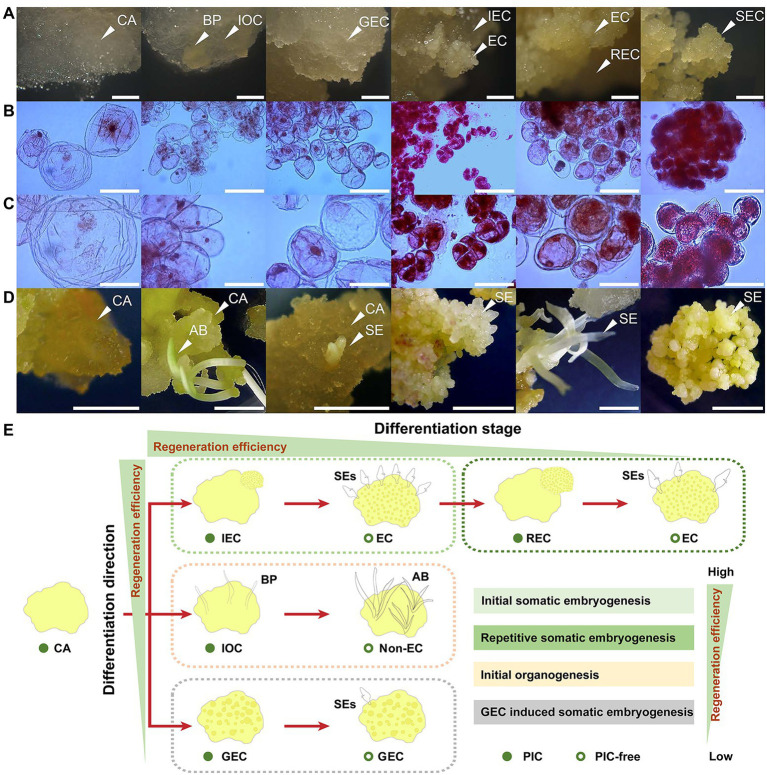
Overview of samples employed in this study. **(A)** Cell morphology and structure of CA, IOC, GEC, IEC, REC, and subcultured-EC (SEC). BP indicates bud primordium; bar represents 1 mm. **(B)** Cell micromorphology of CA and EC; bar represents 100 μm. **(C)** Cell micromorphology of CA and EC; bar represents 50 μm. **(D)** Morphogenesis and regeneration efficiency of CA, IOC, GEC, IEC, REC, and SEC, induced using an auxin-free medium. AB indicates adventitious bud; bar represents 10 mm. **(E)** Model diagram of callus regeneration efficiency in *A. praecox*.

*Agapanthus praecox* is a monocotyledonous, herbaceous, and perennial plant. This species is well known for its ornamental qualities, which include cut flowers, potted plants, and floral border plants in the landscape. In *A. praecox*, the initial flowering takes 3–4 years, and then, flowering occurs annually thereafter. Seed propagation is time-consuming and limited by character segregation in this species. Therefore, it is necessary to establish an efficient regeneration system because of the longer reproductive cycle. To date, the organogenesis and SE pathway were established in *A. praecox* preliminarily. CA was always induced by picloram (PIC), and then the organs (usually buds and roots) and EC could be induced. However, CA cells acquired a specific competence state to achieve regeneration ability (totipotency and pluripotency; Fehér, 2015; Wójcikowska et al., 2020). The organs always regenerated from the interior or surface of the initial organogenesis-based callus (IOC), while initial EC emerged from the surface of the initial embryogenic cell-originated callus (IEC). Besides, repetitive EC was gained from the repetitive embryogenic cell-originated callus (REC). Interestingly, the CA turned into a gradual embryogenic cell-originated callus (GEC), which showed relative opacity and slightly yellowish color, and then induced a few numbers of SEs (Figure 1). However, despite several published studies on SE and organogenesis in *A. praecox*, little information was gained concerning the mechanisms of CA differentiation direction and stage in *A. praecox*. The co-existence of multiple regeneration modes indicated that *A. praecox* was an unusual model to study the mechanism of the acquisition of embryogenic ability in plants.

SEs developed when EC was exposed to exogenous auxin-free conditions (Su et al., 2021), with few barriers to plant regeneration in *A. praecox*. However, the acquisition of EC became a huge difficulty, which showed randomness and inefficiency (Aguilar-Hernández and Loyola-Vargas, 2018). Many plants face a similar challenge, including even some important crops, such as *O. sativa*, *Z. mays*, *G. hirsutum*, and *Triticum aestivum* (Yin et al., 2008; Yang et al., 2014; Guo et al., 2019, 2020; Wang et al., 2022). Therefore, the transition from CA to EC is the key to SE. Also, the lacking of a stable and accurate EC induction method is the bottleneck that restricts the theoretical research and application of SE. Further, the mechanism of embryogenic fate determination during the EC induction process is a key scientific question that has not been addressed yet in many plants (Aguilar-Hernández and Loyola-Vargas, 2018; Wang et al., 2020). The regulatory roles of chromatin accessibility, phytohormones, and stress-related events during SE in *Arabidopsis thaliana* have been well studied (Su et al., 2009; Horstman et al., 2017; Wang et al., 2020). So far, few studies have revealed the mechanisms comprehensively, especially the induction of EC, due to the complexity and individuation of SE (Wang et al., 2022). Proteins and metabolites are known as the active controllers of cellular physiology (Wilinski et al., 2019; Castander-Olarieta et al., 2021). The combination of proteomics and metabolomics can reveal the interactions that initiate the transitions between physiological states (Ribeiro et al., 2019), but the influence of proteins and metabolites related to EC formation in *A. praecox* remains unclear.

The objective of this study was to gain insights into the molecular changes leading to the cell differentiation directions (IEC, IOC, and GEC) and stages (IEC and REC) of *A. praecox*, especially the acquisition of embryogenic ability in *A. praecox*. Detailed knowledge of protein accumulation and metabolite accumulation profiles associated with the embryogenic state may help in improving EC initiation in *A. praecox*. For this, we compared samples including CA and EC originating from a single-cell line and strictly initiated and then proliferated them in the same media and under the same environmental conditions. We applied a novel approach involving integrated multi-scale analyses, combining proteomic and metabolomic profiling, followed by KEGG pathway XML (KGML) analyses to elucidate the differences in functions and interactions of significant factors (proteins and metabolites) related to embryogenic ability determination. We also verified the effects of important pathways on cell differentiation direction, which could improve EC induction specifically in *A. praecox*.

## Materials and Methods

### Plant Material and Culture Conditions

The CA of *A. praecox* was induced using pedicel as explants. CA was induced and transferred monthly on MS medium containing 1.5 mg L^−1^ PIC and 30 g L^−1^ sucrose. CA and EC were cultured in the phytotron in the dark at 25 ± 1°C. Single kind (sucrose, glucose, and maltose) or combination (sucrose and maltose) of carbon sources were employed in the confirmatory experiment. CA was subcultured on MS medium contained 1.5 mg L^−1^ PIC and 30 g L^−1^ carbon source to investigate the effects of sugars on cell differentiation direction. Nine cell masses (approximately 1 g) were planted per Petri dish, and each replicate contained ten Petri dishes, and three biological replicates were conducted. The data was collected after 30 days.

### Morphological Microscopy Observation

For cell morphological observation, CA, EC, and organ morphological image was captured using Stereo Discovery V20 Macro Stereo (Carl Zeiss, Germany). For cell microscopy observation, fresh CA and EC blocks (<5 mm^3^) were transferred to a 1.5-ml Eppendorf tube immediately, and the samples were suspended in 0.5 ml of 1% (*w*/*v*) acetocarmine for 30 min. Then the CA and EC suspension was diluted with ddH_2_O, the suspension including CA and EC cells (<0.5 mm^3^) was transferred to a slide, and a cover glass was placed on the slide slightly. Microscopic observations were performed using an Axio Scope A1 microscope (Carl Zeiss, Germany). The cell diameter was calculated using 10 individual cells.

### Proteome Profiling

Proteins were extracted from three biological replicates per sample, and 0.1 g of the frozen samples were placed in a cold mortar. A mortar and a pestle were used to grind the tissue into a fine powder, and 1 ml of Tris-phenol buffer was added to incubate at room temperature for 10 min. Then, 1 ml of saturated phenol was added, and the mixture was shaken for 40 min at 4°C. The tubes were centrifuged at 5,000 *g* for 15 min at 4°C, and the upper phenolic phase was collected. Subsequently, cold 0.1 M ammonium acetate–methanol solution was added at −20°C using five volumes of the collected phenolic phase. The sediment was collected after centrifugation at 12,000 *g* for 10 min at 4°C. Then, the sediment was collected, repeating this step one more time, and dried at room temperature for 2 min. Subsequently, the sediment was resuspended in 300 μl of lysate solution [8 M urea and 40 mm Tris–HCl containing 1 mM phenylmethylsulfonyl fluoride (PMSF), 5 mm dithiothreitol (DTT), and 2 mm ethylenediaminetetraacetic acid (EDTA)] for 3 h. Finally, the supernatant was the extracted protein solution after centrifugation of mixtures at 12,000 *g* for 10 min at room temperature. The measurements of protein were performed by the Bradford method. TMT labeling was performed according to the FASP method (Jiang et al., 2021). For TMTpro 16 labeling, the lyophilized samples were resuspended in 100 μl of 100 mm TEAB, pH 8.5, and 40 μl of each sample were transferred to new tubes for labeling. Anhydrous acetonitrile was added to the TMT reagent vial at room temperature. The centrifuged reagents were dissolved for 5 min and mixed for centrifugation; this step was repeated once. Then, 10 μl of the TMTpro label reagent was added to each sample for mixing. The tubes were incubated at room temperature for 1 h. Finally, 5 μl of 5% hydroxylamine was added to each sample and incubated for 15 min to quench the reaction. The labeling peptides solutions were lyophilized and stored at −80°C. All samples were analyzed using a Triple TOF 5600 mass spectrometer (SCIEX, United States). The flow rate of the Eksigent nanoLC-1D plus system (SCIEX) was 300 μl/min, and the linear gradient was 90 min (from 5 to 85% B over 67 min; mobile phase A = 2% ACN/0.1% FA and B = 95% ACN/0.1% FA). A rolling collision energy voltage was used for CID fragmentation for MS/MS spectra acquisitions. The mass was dynamically excluded for 22 s. Enrichment analysis of the proteins was performed using the cloud platform of OmicsBean to analyze the functional characteristics of the selected DAPs. The LC–MS/MS raw data were imported in MaxQuant for analysis. The database was offered by Proteome Discover 2.4 (Thermo Fisher). Proteins with |log_2_-fold change| > 1 and *P* adj < 0.05 were considered DAPs. For this analysis, protein IDs assigned was based on the homology of KO (KEGG Orthology), and the online Kyoto Encyclopedia of Genes and Genomes (KEGG)^1^ was used to classify the identified proteins. In addition, non-commercial databases, including metabolite pathways, were searched on KEGG.

### Metabolome Profiling

The fresh samples (80 mg) were transferred to a 1.5-ml Eppendorf tube. Two small steel balls were added to the tube. Further, 360 μl of cold methanol and 40 μl of 2-chloro-l-phenylalanine (0.3 mg/ml) dissolved in methanol as the internal standard were added to each sample. The samples were placed at −80°C for 2 min and ground at 60 Hz for 2 min. The mixtures were ultrasonicated at ambient temperature for 30 min. Then, 200 μl of chloroform was added to the samples, the mixtures were vortexed, and 400 μl of water was added. The samples were vortexed again and then ultrasonicated at ambient temperature for 30 min. They were centrifuged at 13,000 rpm for 10 min at 4°C. The QC sample was prepared by mixing aliquots of all samples to be a pooled sample. An aliquot of the 200-μL supernatant was transferred to a glass sampling vial for vacuum-drying at room temperature. Then, 80 μl of 15 mg/ml methoxylamine hydrochloride in pyridine was subsequently added. The resultant mixture was vortexed for 2 min and incubated at 37°C for 90 min. Next, 80 μl of BSTFA (with 1% TMCS) and 20 μl of n-hexane were added to the mixture, vortexed for 2 min, and then derivatized at 70°C for 60 min. The samples were placed at ambient temperature for 30 min before GC–MS analysis. The derivatized samples were analyzed on an Agilent 7890B gas chromatography system coupled to an Agilent 5977A MSD system (Agilent Technologies Inc., CA, United States). A DB-5MS fused-silica capillary column (30 m × 0.25 mm × 0.25 μm, Agilent J & W Scientific, CA, United States) was used to separate the derivatives. Helium (>99.999%) was used as the carrier gas at a constant flow rate of 1 ml/min through the column. The injector temperature was maintained at 260°C. The injection volume was 1.0 μl in the split-less mode. The initial oven temperature was 60°C, ramped to 125°C at a rate of 8°C/min, to 210°C at a rate of 4°C/min, to 270°C at a rate of 5°C/min, to 305°C at a rate of 10°C/min, and finally held at 305°C for 3 min. The temperature of MS quadrupole and ion source (electron impact) was set to 150, and 230°C, respectively. The collision energy was 70 eV. The mass data were acquired in a full-scan mode (m/z 50–500). ChemStation software (version E.02.02.1431, Agilent, United States) was used to acquire and preprocess the data. Metabolites were annotated based on the Fiehn or NIST databases. After alignment with statistic compare component, the raw data array was obtained from raw data with three dimensions data sets including sample information, peak names, and peak intensities. In the data array, all internal standards and any known pseudo positive peaks were removed. After RSD of the interior label >0.3 deleted, all peak strength (peak area) was processed by normalization of multi-interior label according to retention time partition period. Data were transformed by log_10_, and the generated data matrix was imported into the SIMCA 14.0 software package (Umetrics, Umeå, Sweden). PCA and (orthogonal) partial least squares-discriminant analysis [(O) PLS-DA] were performed to visualize the metabolic difference among experimental groups after mean centering and unit variance scaling. Variable importance in the projection (VIP) ranked the overall contribution of each variable to the OPLS-DA model, and variables with VIP > 1 were considered relevant for group discrimination. Metabolites with *p* ≤ 0.05 and VIP  ≥ 1.0 were considered significant differentially accumulated metabolites (DAMs). KEGG pathway analysis in metabolites was performed by OE Biotech. Co., Ltd. (Shanghai, China).^2^

### Integrated Proteome and Metabolome Analysis

The proteins and corresponding metabolites were considered to be correlated if they were both accumulated in the same sample. Based on the log_2_ fold change of DAPs and DAMs, the Spearman correlation coefficients and associated *p*-values were calculated; the correlation plots of comparative analyses were also drawn. KEGG pathway enrichment analyses were then visualized. Moreover, the cognate DAPs and DAMs were mapped to the reference pathways in the KEGG database to better understand the regulatory status of DAPs and DAMs in pathways.

### Transcriptome Sequencing

Total RNA was extracted using an RNAprep Pure Plant Kit (Tiangen, China). The integrity of the extracted RNA was verified on an Agilent 2,100 Bioanalyzer (Agilent Technologies, CA, United States). The cDNA library construction and transcriptome sequencing were performed by Origin-gene Biotech. Co., Ltd. (Shanghai, China)^3^ on an Illumina HiSeq 2,500 platform, and paired-end reads were generated. The functions of unigenes were annotated based on public databases, including NCBI non-redundant (NR), SwissProt, KEGG pathway, Clusters of Orthologous Groups for eukaryotic complete genomes (KOG), and the GO database using an e-value of 10^−5^. The relationship between the SwissProt and GO terms was mapped for GO classification, based on the SwissProt annotation. The KEGG database was used to annotate potential unigene metabolic pathways. Genes with |log_2_ fold change| > 1 and *P* adj < 0.05 were considered DEGs.

### Statistical Analysis

The data including callus multiplication, cell mass, cell size was statistically tested using one-way analysis of variance with SPSS software (SPSS Inc., IL, United States; v20.0). The significance was shown with different lowercase letters at *p* < 0.05.

## Results

### Overview of Morphological, Proteomic, and Metabolic Analyses for Cell Differentiation

Samples with different differentiation directions and stages are demonstrated in Figure 1A. The CA was usually whitish, semitransparent, surfacing, and watery. The IOC showed similar texture and color with the CA, but it could generate a bud primordium (BP), which was opaque and granular. The GEC was relatively opaque and slightly yellowish compared with the CA, and with a rough surface. The IEC generated small clumps and a compact, bright yellow cell mass, which showed a high proliferation rate and cell viability. Also, cell divisions were usually more frequent in embryogenic lines, which grew more rapidly than in non-EC. Accordingly, the microscopic comparison revealed large numbers of mitotic meristematic cells in IEC (Figures 1B,C). REC produced loose, yellow clumps of cells. SEC was friable in texture, rough in surface, and yellow in color. Non-EC and EC were demonstrated clearly by acetocarmine staining. The CA, IOC, and GEC had large cell diameters and thin cytoplasm, while the IEC, REC, and SEC had small cell diameters and dense cytoplasm. Interestingly, the cell size of EC generated from the IEC was significantly reduced compared with that of the CA (Figures 1B,C). Samples related to different differentiation directions and stages showed widely different regeneration efficiency when cultured on a PIC-free medium. The CA tended to be light brown because of the absence of exogenous auxin, and adventitious buds and SE were induced from the IOC and IEC, respectively. A few numbers of SEs were induced in the sample of the GEC. The REC regenerated SE spontaneously, but it was accompanied by lower SE efficiency. Most of the SEs showed an abnormal polarity structure in REC. The SEC induced SEs naturally, but the SE number decreased appreciably compared with those induced by the IEC (Figure 1D). The regeneration efficiency showed huge differences among the samples, which indicated that IEC is the ideal model for plant regeneration *in intro* in *A. praecox* (Figure 1E).

**Figure 2:**
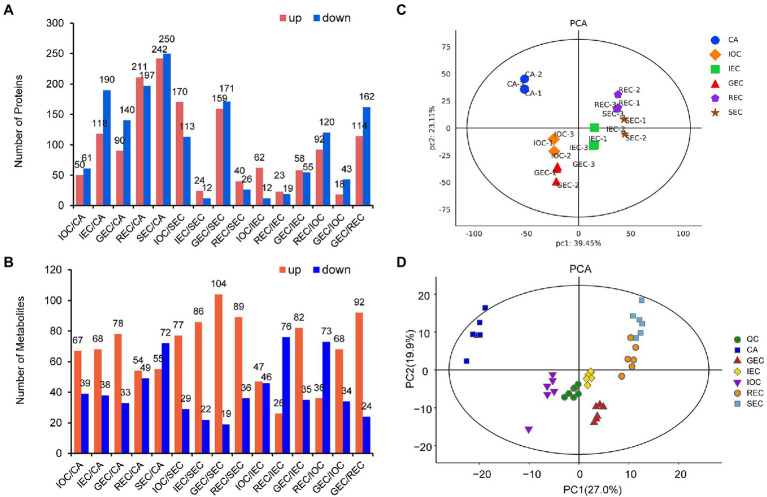
Overview of proteomic and metabolomic differences between samples. **(A)** Numbers of DAPs in different comparisons. **(B)** Numbers of DAMs in different comparisons. **(C)** PCA model of proteomics. **(D)** PCA model for metabolomics.

The samples were divided into two groups by TMTpro 16 labeling quantitative proteomics. The quantitative analysis showed that 12,509 unique peptides corresponding to 4,221 proteins were identified in group 1, and 12,555 unique peptides corresponding to 4,203 proteins were authenticated in group 2. We identified 36–492 DAPs between different comparison groups. The comparison group of SEC/CA showed huge differences, while the comparison group of IEC/SEC showed little differences (Figure 2A). The statistical analysis of metabolites showed that the DAMs ranged from 93 to 127 between different comparison groups. The comparison group of SEC/CA showed huge differences, while the comparison group of IOC/IEC showed little differences (Figure 2B).

The principal component analysis (PCA) model of proteomics on our paired datasets showed a high concordance between three replicates. Samples from different differentiation directions and stages were separated by PC1 (39.45%) and PC2 (23.11%). CA was more dispersive than the other samples, implying that protein accumulation patterns varied during CA differentiation. In addition, non-EC samples from different groups could be distinctly separated by PC1, indicating that EC and non-EC exhibited accumulation variation at the same stage (Figure 2C). We mixed (in equal proportions) samples to create QC samples to test the stability of the mass spectrometry system. The PCA model diagram showed that the QC samples were closely clustered, indicating that the experiment was stable and repeatable (Figure 2D). The PCA model for metabolomics on our paired datasets showed high concordance between six replicates. In addition, non-EC samples from different groups could be distinctly separated by PC1 (27.0%), while the initial differentiation samples (IOC, IEC, and GEC) were distinctly separated by PC2 (19.9%). The results indicated that the PCA analysis of metabolites was highly consistent with the PCA model in proteomic profiles.

The results indicated that CA samples underwent complex diversity concerning cell differentiation. Interestingly, morphological, proteomic, and metabolomics analyses could clearly distinguish the direction and stage of cell differentiation in *A. praecox*.

### Proteomic Analyses of Cell Differentiation

**Figure 3:**
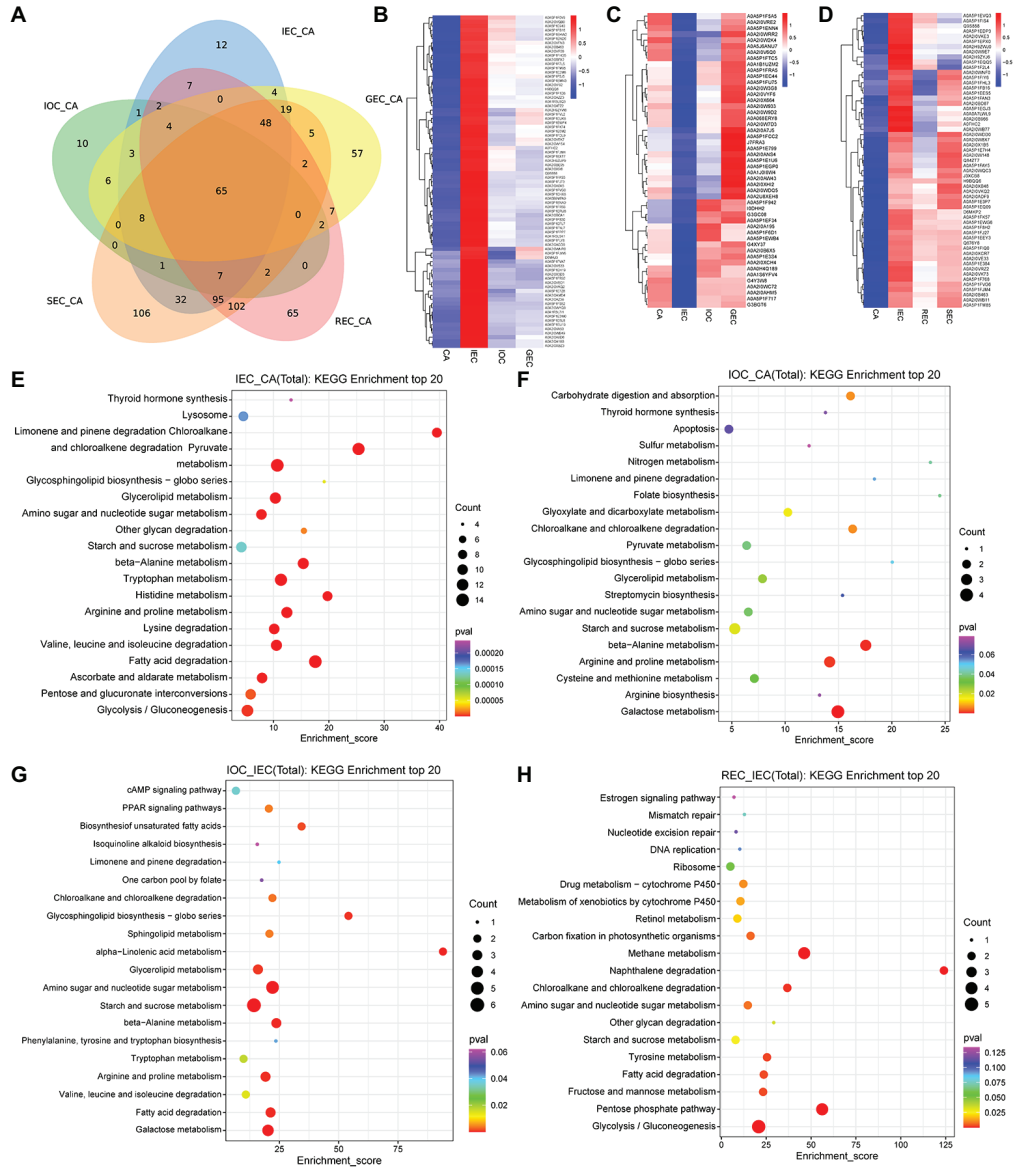
Accumulation analysis and quantitative comparison of DAPs with different directions and stages of cell differentiation in *A. praecox*. **(A)** Venn diagram of DAPs among five compared pairs (IEC/CA, IOC/CA, GEC/CA, REC/CA, and SEC/CA). **(B)** Hierarchical clustering analysis (HCA) of DAPs concerning CA differentiation direction; the cluster showed DAPs with higher accumulation in IEC. The horizontal axis represents the sample clusters, and colors from blue to red indicate protein accumulation from low to high, the same below. **(C)** HCA of DAPs concerning CA differentiation direction; the cluster showed DAPs with lower accumulation in IEC. **(D)** HCA of DAPs concerning CA differentiation stage. **(E)** KEGG pathway enrichment of the comparative analyses in IEC/CA. The enrichment score is the percentage of members out of the total number detected. The bubble size represents the number of members detected in the KEGG pathway, and the color of the bubble represents the *p*-value, the same below. **(F)** KEGG pathway enrichment of the comparative analyses in IOC/CA. **(G)** KEGG pathway enrichment of the comparative analyses in IOC/IEC. **(H)** KEGG pathway enrichment of the comparative analyses in REC/IEC.

Venn diagram shows shared and unique DAPs among five compared pairs (IEC/CA, IOC/CA, GEC/CA, REC/CA, and SEC/CA). In total, 672 proteins were significantly differentially accumulated between CA and EC of all six samples. Among these, 12 DAPs were strongly accumulated especially in IEC, 10 DAPs were accumulated specifically in IOC, and 65 were accumulated in REC (Figure 3A).

DAPs were analyzed by hierarchical clustering using CA, IEC, IOC, and GEC as samples to identify the differentiation direction of CA. The K-means clustering approach based on the level of protein accumulation was used to sort all differential peaks resulting in 10 clusters, named C1 to C10, respectively (Supplementary Figure S1). As illustrated in Figure 3B, 79 DAPs (C7) were over-accumulated in IEC. As shown in Figure 3C, 49 DAPs (C2) were down-accumulated in IEC. Similarly, differentiation stage-specific patterns were present according to their relative abundance, and the number ascribed to each cluster was also recorded (Supplementary Figure S2). The results indicated that DAPs accumulated in C5 were specifically accumulated in IEC, comprising 57 proteins showing a marked increase from CA to IEC (Figure 3D).

The proteomic results *via* KEGG analysis revealed that different pathways were enriched concerning cell differentiation. A total of 126 pathways were enriched in comparing the IEC/CA (Supplementary Table S1). These DAPs were involved mainly in pathways (level 2) including the metabolism of terpenoids and polyketides, xenobiotics biodegradation and metabolism, amino acid metabolism, glycan biosynthesis and metabolism, lipid metabolism, metabolism of other amino acids, endocrine system, metabolism of cofactors and vitamins, and carbohydrate metabolism. The top 20 terms are shown in Figure 3E. Similarly, 61 pathways were enriched in the comparison of IOC/CA (Supplementary Table S2). We observed that DAPs were involved mainly in pathways (level 2) including metabolism of cofactors and vitamins, energy metabolism, glycan biosynthesis and metabolism, metabolism of terpenoids and polyketides, metabolism of other amino acids, xenobiotics biodegradation and metabolism, digestive system, biosynthesis of other secondary metabolites, carbohydrate metabolism, amino acid metabolism, endocrine system, and lipid metabolism. The top 20 terms are shown in Figure 3F. A total of 42 pathways were enriched between the IOC and IEC (Supplementary Table S3). DAPs were involved mainly in the pathways (level 2) including lipid metabolism, glycan biosynthesis and metabolism, metabolism of terpenoids and polyketides, metabolism of other amino acids, amino acid metabolism, carbohydrate metabolism, xenobiotic biodegradation and metabolism, endocrine system, metabolism of cofactors and vitamins, biosynthesis of other secondary metabolites, and energy metabolism. The top 20 terms are shown in Figure 3G.

Among DAPs, top DAPs were enriched in the comparison groups of IEC/CA, IOC/CA, and IOC/IEC were listed in Supplementary Table S4. Among them, peroxidase (A0A5P1F2L4), C2H2-type domain-containing protein (A0A5P1FM85), and Histone H2B (A0A5P1FK74) were over-accumulated significantly in IOC and IEC compared to CA. Meanwhile, BTB/POZ domain-containing protein (A0A2H9ZSK5), peroxidase (A0A2I0X575), and Beta-D-xylosidase (A0A2I0V9J4) were over-accumulated considerably in IOC compared to IEC. The results above indicated that carbohydrate and energy metabolism, amino acid metabolism, secondary metabolites, chromatin accessibility, and DNA methylation may play important roles in cell differentiation.

We screened 27 pathways enriched in comparing the REC/IEC (Supplementary Table S5). DAPs were involved in xenobiotic biodegradation and metabolism, carbohydrate metabolism, energy metabolism, glycan biosynthesis and metabolism, amino acid metabolism, lipid metabolism, energy metabolism, replication and repair, xenobiotic biodegradation and metabolism, metabolism of cofactors and vitamins, endocrine system, and aging. The top enriched pathways are shown in Figure 3H, revealing that carbohydrate and energy metabolism, aging, and genetic information processing were changed remarkably by cell differentiation stage.

### Metabolomic Analyses of Cell Differentiation

**Figure 4:**
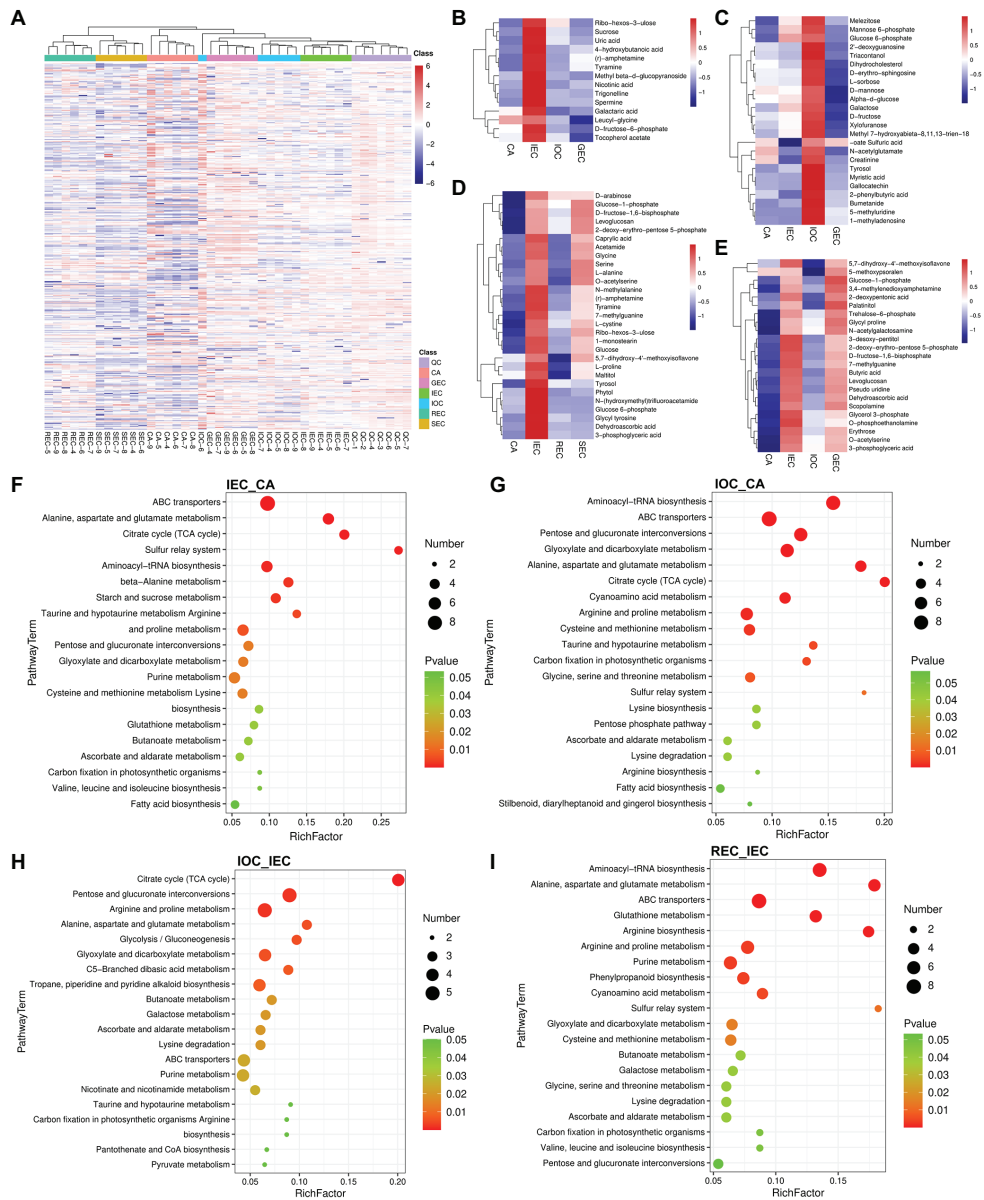
Hierarchical clustering analyses and comparison of DAMs in the direction and stage of cell differentiation in *A. praecox*. **(A)** HCA among samples. The horizontal axis represents the sample clusters, and colors from blue to red indicate metabolite accumulation from low to high, the same below. **(B)** HCA of DAMs concerning CA differentiation direction, and the cluster showed DAMs with higher accumulation in IEC. **(C)** HCA of DAMs concerning CA differentiation direction, and the cluster showed DAMs with higher accumulation in IOC. **(D)** HCA of DAMs concerning CA differentiation stage. **(E)** HCA of DAMs concerning CA differentiation direction. **(F)** KEGG pathway enrichment of the comparison of IEC/CA. The bubble size represents the number of members detected in the KEGG pathway, and the color of the bubble represents the *p*-value, the same below. **(G)** KEGG pathway enrichment of the comparison of IOC/CA. **(H)** KEGG pathway enrichment of the comparison of IOC/IEC. **(I)** KEGG pathway enrichment of the comparison of REC/IEC.

Hierarchical clustering for the enriched levels of all metabolites intuitively demonstrated the stability of the relationship between QC and other samples. It showed that samples including GEC, IOC, and IEC were grouped together, REC and SEC were gathered in another cluster, and the two groups were separated from CA (Figure 4A).

In this study, 390 DAMs were identified, which mainly included sugars, glucosides, amino acids, organic acids, and secondary metabolites. DAMs were analyzed by hierarchical clustering based on the accumulation of significantly different metabolites in each group to select marker metabolites accurately and investigate changes in the related metabolic processes (Supplementary Figure S3). A clear separation of metabolites existed between the over-accumulated and down-accumulated ones with the comparison of direction with cell differentiation.

DAMs were analyzed by hierarchical clustering using CA, IEC, IOC, and GEC as samples to identify the metabolic profile of cell differentiation direction. K-means clustering approach based on the level of metabolites accumulation was used to sort all differential peaks, resulting in eight clusters, named C1 to C8 (Supplementary Figure S4). As illustrated in Figure 4B, DAMs including sucrose, D-fructose-6-phosphate, and galactaric acid were highly accumulated in IEC. As shown in Figure 4C, DAMs including mannose 6-phosphate, glucose 6-phosphate, L-sorbose, d-mannose, galactose, and D-fructose were accumulated highly in IOC. Besides, DAMs including glucose-1-phosphate, trehalose-6-phosphate, 3-desoxy-pentitol, D-fructose-1,6-bisphosphate, and levoglucosan were highly accumulated in IEC and GEC (Figure 4E). Differentiation stage-specific patterns were present according to their relative abundance. The DAMs were also screened by the differentiation stage. Broken line graphs were drawn to classify the accumulation patterns of DAMs, and the number ascribed to each cluster was also recorded (Supplementary Figure S5). The results indicated that DAMs accumulated in C8 were specifically accumulated in IEC samples; 29 DAMs showed a marked increase from CA to IEC. Further, these DAMs were specifically accumulated in IEC than in REC and SEC. The DAMs were enriched in the carbohydrate metabolism pathway, including D-arabinose, glucose-1-phosphate, D-fructose-1,6-bisphosphate, ribo-hexos-3-ulose, glucose, maltitol, and glucose 6-phosphate (Figure 4D). Notably, dehydroascorbic acid was enriched in IEC, not only in the analyses of differentiation direction but also in the analyses of differentiation stage (Figures 4D,E). Interestingly, most of the CA metabolites identified during different directions and stages were enriched in the same pathways, such as sugar metabolism, arginine and proline metabolism, galactose metabolism, glyoxylate and dicarboxylate metabolism, and ascorbate and aldarate metabolism (Figures 4D,E).

All of the DAMs differing by cell differentiation were mapped to the KEGG database to analyze the relevant pathways. The results showed that 60, 60, 54, and 62 pathways were enriched in DAMs in the comparison groups of IEC/CA, IOC/CA, IOC/IEC, and REC/IEC, respectively (Supplementary Table S6). The top 20 metabolic pathways with significant enrichment were selected for bubble mapping to understand the differential metabolic pathways of various samples by comparing DAMs in the KEGG database. The CA differentiation-related pathways were enriched as follows: ABC transporters, alanine, aspartate and glutamate metabolism, citrate cycle (TCA cycle), sulfur relay system, aminoacyl-tRNA biosynthesis, taurine and hypotaurine metabolism, arginine and proline metabolism, pentose and glucuronate interconversions, glyoxylate and dicarboxylate metabolism, purine metabolism, lysine biosynthesis, ascorbate and aldarate metabolism, carbon fixation in photosynthetic organisms, and fatty acid biosynthesis (Figure 4). The changes in these metabolites and metabolic pathways provided important information on how *A. praecox* responded to cell differentiation. Interestingly, the pathways including beta-alanine metabolism, starch and sucrose metabolism, cysteine and methionine metabolism, glutathione metabolism, and ascorbate and aldarate metabolism were specific in IEC/CA (Figure 4F). The pathways including cyanoamino acid metabolism, serine and threonine metabolism, pentose phosphate pathway, arginine biosynthesis, and stilbenoid, diarylheptanoid, and gingerol biosyntheses were specific in IOC/CA (Figure 4G). Besides, DAMs related to tropane, piperidine, and pyridine alkaloid biosynthesis, butanoate metabolism, galactose metabolism, nicotinate and nicotinamide metabolism, pyruvate metabolism were differentially accumulated in IOC/IEC (Figure 4H). The pathways including glutathione metabolism, phenylpropanoid biosynthesis, cysteine and methionine metabolism, butanoate metabolism, galactose metabolism, and valine, leucine, and isoleucine biosyntheses were significantly changed in the REC compared with the IEC (Figure 4I). Besides, plant hormones including auxin and cytokinin biosynthesis-related pathways, such as tryptophan metabolism, and zeatin biosynthesis were screened (Supplementary Table S6).

Top DAMs screened in the comparison groups of IEC/CA, IOC/CA, and IOC/IEC were listed in Supplementary Table S7. Among them, 3,4-methylenedioxyamphetamine, glucose, glycyl tyrosine sedoheptulose, lactitol, and chlorogenic acid were accumulated significantly different in the IOC compared to CA. Meanwhile, the accumulation of butyric acid, scopolamine, 3,4-methylenedioxyamphetamine, ribo-hexos-3-ulose, and glucose showed significant differences in the IEC compared to CA. Interestingly, the accumulation of glucose showed 821.098 and 772.350 times in the IOC and IEC compared to CA, respectively. The top DAMs enriched suggested carbohydrate and energy metabolism was changed remarkably in cell differentiation.

### Integrated Proteomic and Metabolomic Analyses of Cell Differentiation

**Figure 5:**
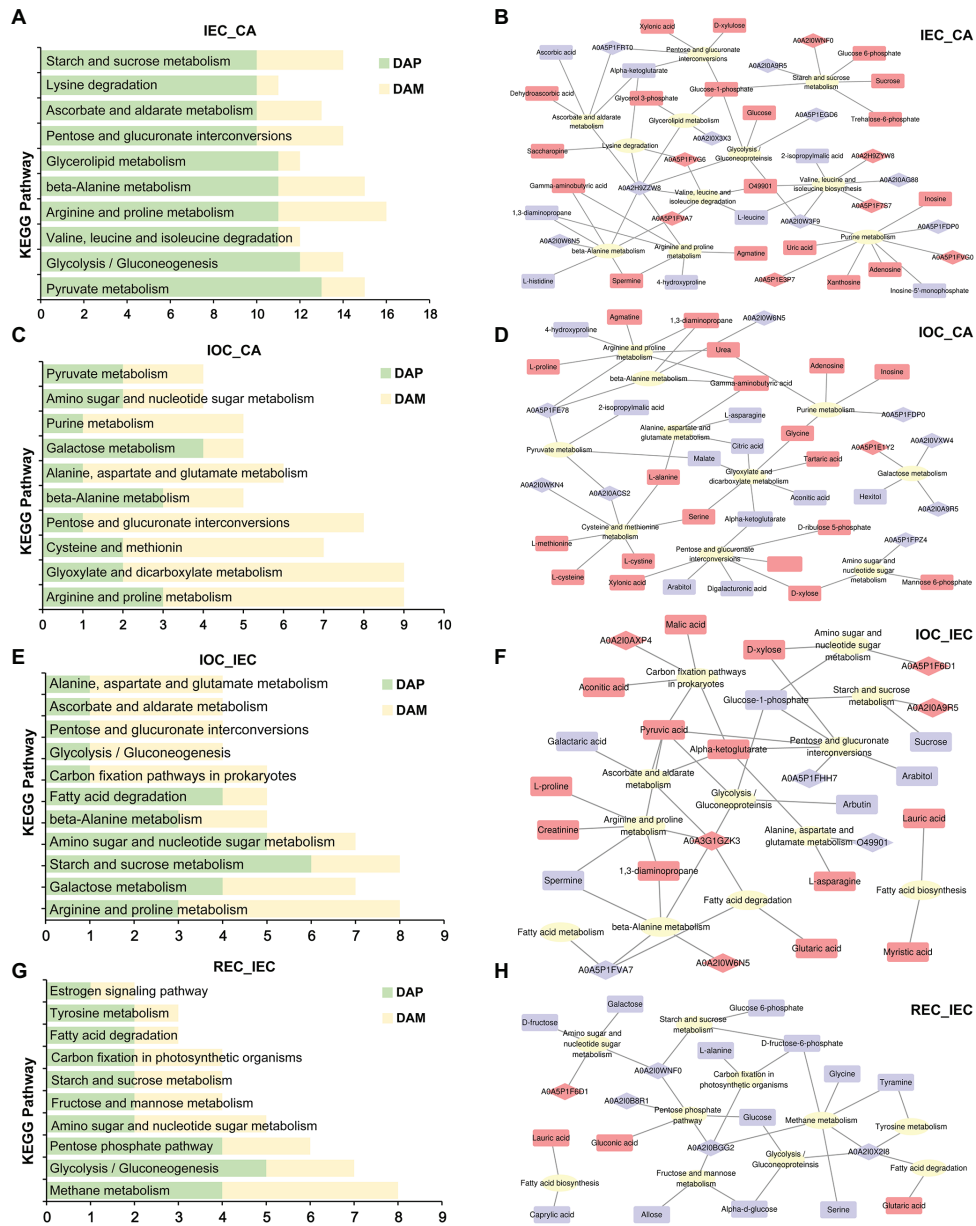
Pathway enrichment of DAPs/DAMs and KEGG pathway XML (KGML) analyses on cell differentiation. **(A)** Pathway enrichment in IEC/CA; numbers presented the amounts of DAPs and DAMs, herein after. **(B)** KGML network of DAPs and DAMs in IEC/CA comparison. The ellipse, diamond, and chamfered rectangle indicate pathways, proteins, and metabolites, respectively. Red and blue indicate DAPs and DAMs over-accumulated or down-accumulated, herein after. **(C)** Pathway enrichment in IOC/CA comparison. **(D)** KGML network of DAPs and DAMs in IOC/CA comparison. **(E)** Pathway enrichment in IOC/IEC comparison. **(F)** KGML network of DAPs and DAMs in IOC/IEC comparison. **(G)** Pathway enrichment in REC/IEC comparison. **(H)** KGML network of DAPs and DAMs in REC/IEC comparison.

We performed a KEGG mapping analysis based on the dataset of DAPs and DAMs. Top 10 pathways containing DAPs and DAMs in different comparisons were listed separately (Figure 5). Further analysis of these pathways suggested that these proteins and metabolites were involved mainly in energy metabolism as well as in fatty acid degradation, pyruvate metabolism, glycolysis/gluconeogenesis, pentose and glucuronate interconversions, starch and sucrose metabolism, galactose metabolism, carbon fixation pathways in prokaryotes, carbohydrate digestion and absorption, and fructose and mannose metabolism and ROS responses and resistance, such as ascorbate and aldarate metabolism (Figures 5A,C,E,G).

We constructed an interactive diagram concerning IEC induction using an integrated analysis of DAPs and DAMs enriched in top 10 KEGG pathways. In the network, starch and sucrose metabolism pathway was enriched, and the accumulation of sugars and their intermediate products, including sucrose, glucose 6-phosphate, glucose-1-phosphate, and trehalose-6-phosphate, was enhanced significantly. Meanwhile, glucose-6-phosphate isomerase (A0A2I0WNF0) was over-accumulated and alpha-glucosidase (A0A2I0A9R5) was down-accumulated. Glucose-1-phosphate played critical roles in the connection between the pathways including starch and sucrose metabolism, glycolysis/gluconeoproteinsis, glycerolipid metabolism, pentose and glucuronate interconversions in amino acid metabolism, and ascorbate and aldarate metabolism, accompanied by the high accumulation of glycerol 3-phosphate, glucose, D-xylulose, and xylonic acid. The metabolite glucose-1-phosphate might interact with several metabolic pathways in the integrated network (Figure 5B).

In the IOC induction network, the enrichment of alpha-ketoglutarate, D-xylose, D-ribulose 5-phosphate, D-xylulose, xylonic acid, arabitol, and digalacturonic acid changed significantly in the pentose and glucuronate interconversions pathway. Meanwhile, hexitol, alpha-glucosidase (A0A2I0A9R5), and beta-glucosidase (A0A2I0VXW4) were suppressed in the galactose metabolism. Mannose 6-phosphate and D-xylose were enhanced in amino sugar and nucleotide sugar metabolism. Besides, common metabolism pathways, such as arginine and proline metabolism, were regulated in the IOC induction process (Figure 5D).

Comparing IOC induction with IEC induction, pathways related to energy metabolism as well as fatty acid biosynthesis, fatty acid degradation, fatty acid metabolism, glycolysis gluconeogenesis, pentose and glucuronate interconversions, starch and sucrose metabolism, carbon fixation pathways in prokaryotes, and amino sugar and nucleotide sugar metabolism were connected. The enrichment pattern indicated that energy metabolism might play key roles in cell differentiation directions. The metabolites including D-xylose, pyruvic acid, alpha-ketoglutarate, malic acid, aconitic acid, alpha-ketoglutarate, lauric acid, myristic acid, and glutaric acid accumulated in IOC compared with IEC. However, the products, such as sucrose, glucose-1-phosphate, and arabitol decreased (Figure 5F).

Comparing REC induction with IEC induction, pathways related to energy metabolism as well as fatty acid biosynthesis, fatty acid degradation, glycolysis/gluconeogenesis, pentose phosphate pathway, starch and sucrose metabolism, carbon fixation pathways in prokaryotes, and amino sugar and nucleotide sugar metabolism were connected. A majority of the DAPs and DAMs showed suppression in the regulated network. Metabolites including alpha-D-glucose, galactose, D-fructose, D-fructose-6-phosphate, allose, glucose, and glucose 6-phosphate decreased remarkably in the REC induction compared with IEC induction. The enrichment pattern indicated that sugar metabolism might play key roles in cell differentiation stages (Figure 5H).

**Figure 6:**
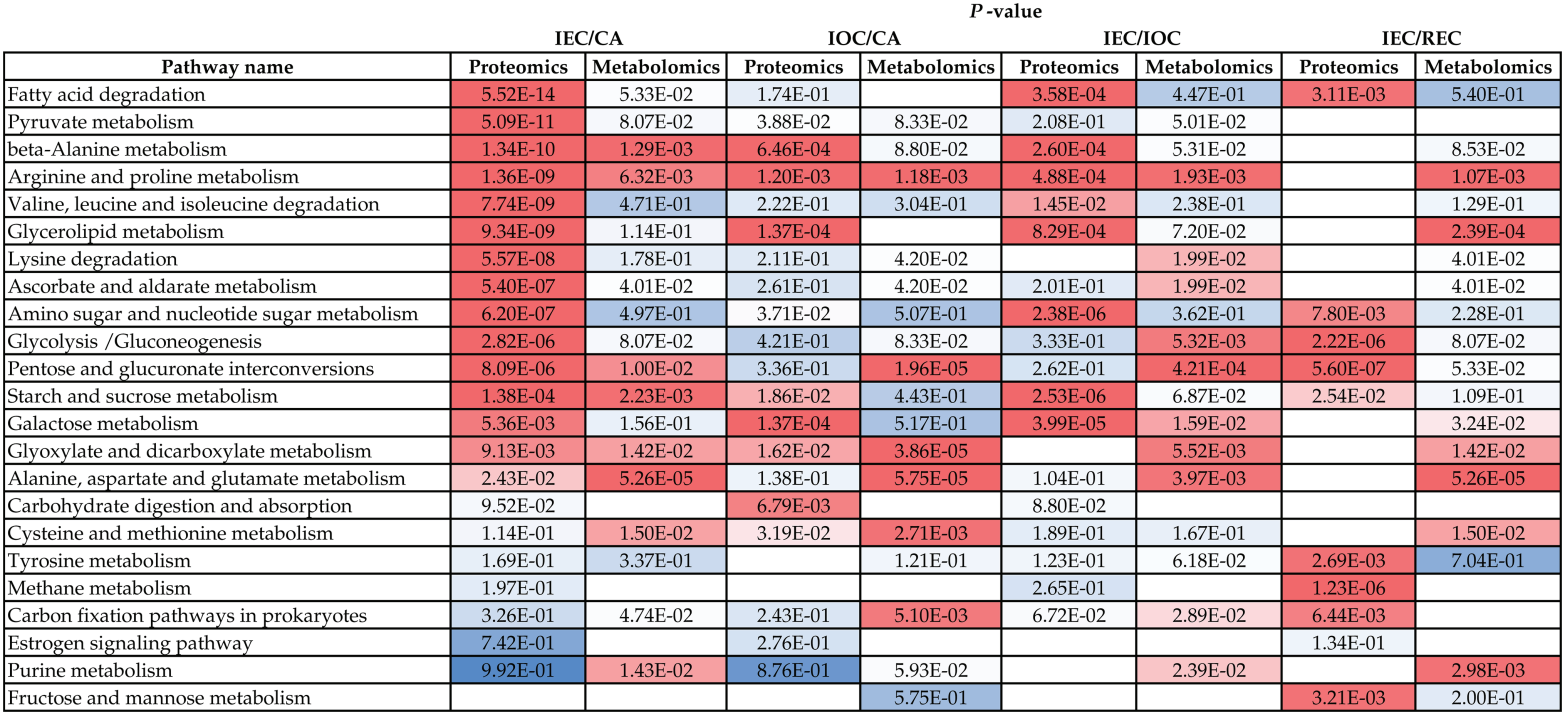
Proteins and metabolites involved in common pathways related to cell differentiation. Data are *p*-values. Colors from red to blue indicate the *p*-value from low to high, and blank space mean not being enriched in KEGG pathways.

By conducting a KEGG mapping analysis based on the DAPs and DAMs, we found that cell differentiation was associated with 23 metabolic pathways (Figure 6). Integrated proteomic and metabolomic pathway analyses suggested that the regulation of sugar metabolism might affect cell differentiation direction and stage in *A. praecox*. The accumulation of A0A2I0WNF0 (glucose-6-phosphate isomerase), glucose-1-phosphate, glucose 6-phosphate, trehalose-6-phosphate, and sucrose, which were involved in starch and sucrose metabolism, was over-accumulated. Notably, alpha-glucosidase was down-accumulated (Supplementary Figure S6). The levels of beta-D-glucose and alpha-D-glucose 1p increased by as much as 9.59 (log_2_ FC) and 1.97 (log_2_ FC), respectively, in the glycolysis/gluconeogenesis pathway (Supplementary Figure S7).

### Effects of Exogenous Carbon Sources on Cell Differentiation

**Figure 7:**
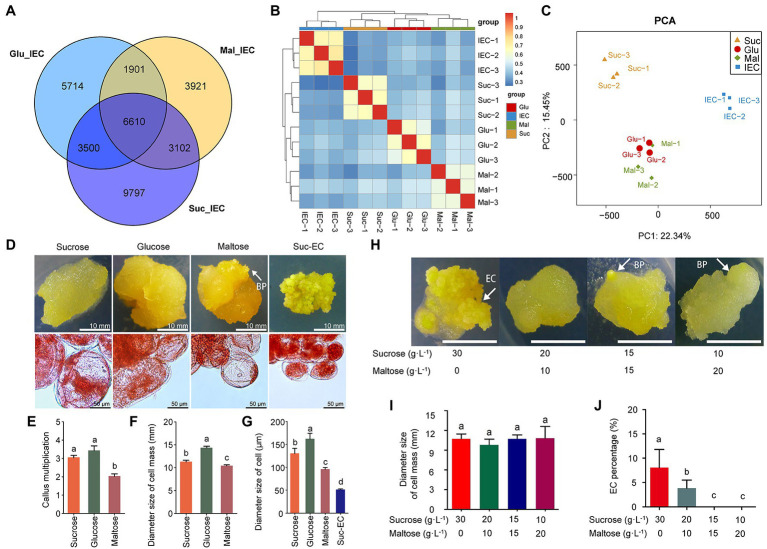
Effects of exogenous carbon sources on cell differentiation. **(A)** Venn diagram of DEGs among three compared pairs (Glu/IEC, Suc/IEC, and Mal/IEC); **(B)** Correlation analysis among samples; the horizontal axis represents the sample clusters, and colors from blue to red indicate the correlation index from low to high. **(C)** PCA model of transcriptomics. **(D)** Morphology of the callus and cells incubated with different carbon sources; cell morphology detected by acetocarmine staining. EC was incubated on a medium including sucrose as the carbon source. **(E)** Statistical analysis of CA multiplication, *n* = 3. **(F)** Statistical analysis of cell mass size, *n* = 3. **(G)** Statistical analysis of single-cell size, *n* = 3. **(H)** Cell differentiation differed by carbon source combination of sucrose and maltose. **(I)** Statistical analysis of cell mass size, *n* = 3. **(J)** EC induction rate by the effects of carbon sources, *n* = 3. Data on the bars marked without the same lowercase letter indicate significant differences at *p* < 0.05.

We compared the effects of the exogenous carbon sources on cell differentiation to explore the effects of sugars metabolism in cell differentiation regulation. The transcriptomic data showed that 9,797, 5,714, and 3,921 DEGs were specifically expressed with sucrose, glucose, and maltose treatment, separately (Figure 7A). The PCA model showed that non-EC (Suc, Glu, and Mal) and IEC samples could be separated distinctly (22.34%), and the CA cultured with sucrose had a closer relationship with the IEC (Figures 7B,C). The transcriptomic results *via* KEGG analysis revealed that different pathways were enriched concerning cell differentiation. Interestingly, plant hormone signal transduction, glycolysis/gluconeogenesis, flavonoid biosynthesis, and starch and sucrose metabolism showed significantly different expression patterns when callus cultured with different carbon sources. The top terms are shown in Supplementary Figure S8. The results indicated that carbon sources might affect EC induction. Subsequently, we incubated the CA with different carbon sources (Figure 7D). The results showed that glucose increased cell multiplication, cell mass size, and cell size, while maltose decreased growth coefficient, cell mass size, and cell size significantly. Maltose promoted organogenesis, and BP was induced evidently (Figures 7E–G). Meanwhile, EC was acquired with sucrose treatment. Further, we found that the proportion of different carbon sources (sucrose and maltose) affected the direction of cell differentiation, sucrose was beneficial to EC induction, and maltose was beneficial to organogenesis (Figures 7H–J). The results indicated that sugar metabolism affected CA differentiation direction in *A. praecox*, exogenous sucrose was indispensable in the process of the acquisition of embryogenic ability, and CA incubated with maltose tended to induce organogenesis.

## Discussion

SE is a powerful tool for plant propagation (De-la-Peña et al., 2015; Ribeiro et al., 2019), particularly cultivars in perennial ornamental species, such as *A. praecox*. We performed proteomic and metabolomic profiling, which could provide important insights into proteins and metabolites accumulation, and identified several SE-related pathways during EC formation in this species. Different numbers of DAPs and DAMs were specifically accumulated in IEC, IOC, and REC, suggesting that these proteins and metabolites contributed to cell differentiation in *A. praecox*. Prominent correlations between EC-specific pathways indicated the possible regulatory roles of carbohydrate and energy metabolism, reactive oxygen species (ROS) responses and resistance, chromatin accessibility and DNA methylation, and plant hormone signals (Aguilar-Hernández and Loyola-Vargas, 2018; Ribeiro et al., 2019; Guo et al., 2020).

### Carbohydrate and Energy Metabolism

Integrated proteomic and metabolomic analyses of cell differentiation with different EC formation capabilities indicated that carbohydrate and energy metabolism might affect cell differentiation in plant regeneration (Ge et al., 2017; Aguilar-Hernández and Loyola-Vargas, 2018). Sugar and energy metabolism played key roles in embryogenic development by supporting cell division and modification (Aguilar-Hernández and Loyola-Vargas, 2018). Proteins and metabolites were accumulated differentially between IEC and IOC. Consistently, the content of glucose was higher in IEC and IOC than CA (Supplementary Table S7). The results suggested an increasing in reducing sugars, such as glucose supported the high frequencies in cell division and differentiation (Ribeiro et al., 2019). Sucrose and maltose in the culture medium significantly influenced EC induction and organogenesis induction, respectively, in *A. praecox*. Interestingly, the content of sucrose was higher in the IEC than IOC (Figure 4B). The results suggested that carbohydrate and energy metabolism played fundamental and significant roles in cell differentiation direction. Although carbohydrate metabolism has previously been reported to be involved in SE, the role of these proteins and metabolites is species-dependent (Aguilar-Hernández and Loyola-Vargas, 2018).

Intracellular sugar metabolism was significantly affected by the carbon sources of culture medium with *in vitro* culture. Plant cells can be adjusted in feedback according to the demand of sugar metabolism. However, carbon sources including sucrose, glucose, fructose, and maltose in the medium had a direct influence on cell division and differentiation (Blanc et al., 2002), suggesting important roles of exogenous carbon sources in cell proliferation and differentiation (Ribeiro et al., 2019).

Sugars play roles in respiration, cellular carbon skeleton, osmotic regulation, and signal transduction in plants (Blanc et al., 2002). Our metabolite–protein correlation network incorporated proteins and metabolites that were differentially accumulated in cell differentiation direction. Primary correlations were found between sugar metabolism, energy and substrate-related proteins (alpha-mannosidase, sucrose synthase, alpha-mannosidase, alpha-galactosidase, NADPH dehydrogenase, beta-galactosidase, pyruvate decarboxylase, glucose-6-phosphate isomerase, and so on), and metabolites (glucose, D-xylulose, D-fructose-1,6-bisphosphate, maltotriose, sucrose, and so on). The metabolic analysis, which indicated sucrose hydrolysis activity and energy status, was higher in IEC than in CA (Aguilar-Hernández and Loyola-Vargas, 2018). This pattern confirmed a need for an increase in reducing sugars during EC induction and multiplication, to support the high frequencies of cell divisions. Notably, we also found that glucose-1-phosphate was a node connecting pathways, such as starch and sucrose metabolism, pentose and glucuronate interconversions, glycerolipid metabolism, and glycolysis/gluconeogenesis. It suggested the activation of energy metabolism in the initial stages, reflecting the need for a large amount of energy to sustain cell division (Ribeiro et al., 2019). Further, we observed that 2-oxoglutarate, which linked the TCA cycle with amino acid, glucosinolate, flavonoid, alkaloid, and gibberellin (GA) biosynthesis (Araújo et al., 2014), specifically accumulated in CA with 2.67 (log_2_ FC) increase in activities compared with IEC. Differential accumulation of 2-oxoglutarate between CA and IEC indicated that cell differentiation was affected not only by the TCA cycle but also by amino acid and hormone metabolism. Altogether, sugars were critical in controlling the transition between CA, IOC, and IEC, potentially by regulating basal metabolism (Aguilar-Hernández and Loyola-Vargas, 2018). Thus, our results suggested that carbohydrate and energy metabolism cooperatively contributed to the activation of EC formation.

### ROS Responses and Resistance

A complex balance exists between intracellular stress signals, hormone signals, and carbohydrate metabolism (Pasternak et al., 2007; Wójcikowska et al., 2020). ROS are readily detectable in plant responses to stresses (Fehér, 2015; Zhu, 2016). The generation and clearance of intracellular ROS are in a dynamic change manner. As signal molecules affect cell proliferation and differentiation, antioxidant enzymes and metabolic enzymes are used for intracellular ROS clearance (Ribeiro et al., 2019). Ascorbate peroxidases (APX) play an important role in ROS metabolism (Zhou et al., 2016; Huang and Li, 2020). Our results showed a correlation between ascorbate and aldarate metabolism and cell status. L-dehydroascorbate accumulation was significantly increased 1.8569 (log_2_ FC) in the IEC than in the CA, reflecting the important role of ROS scavenging in EC competence acquisition (Fehér, 2015). The levels of antioxidative proteins, such as APX and glutathione-S-transferase, are frequently higher in the IEC than in the CA; this has been regarded as a determinant of cell embryogenic capacity (Zhou et al., 2016; Huang and Li, 2020). However, glutathione-S-transferase also participates in cell signaling, catalase in sugar or amino acid metabolism, and ascorbate peroxidase in the glutathione-ascorbate cycle; all were activated in embryogenic tissues (Żur et al., 2019). Thus, these enzymes may contribute to embryogenic capacity through various developmental and cell cycling processes rather than merely through the deactivation of ROS (Zhou et al., 2016). Moderate-intensity ROS, such as H_2_O_2_, usually promotes the *in vitro* culture and redifferentiation of plant cells (Zhou et al., 2016), while excess ROS cause membrane lipid peroxidation and lead to cell electrolyte disorder (Zeng et al., 2017). On the contrary, stress upregulated the expression of modifying enzymes, such as expansins and xyloglucan, that remodeled the cell wall (Zhu, 2016), which ultimately affected cell enlargement and division. These stress defense reactions have been interpreted as responses of the tissues to *in vitro* culture conditions (Zhao et al., 2015). They are probably not directly related to a specific morphogenic pathway, such as SE, but may be prerequisites for embryogenesis. Indeed, the proliferation and differentiation of cell clusters provided the basis of regeneration, and strong resistance for ROS provided the possibility of regeneration. Our result confirmed that defense responses against abiotic stress modulated embryogenic capacity (Fehér, 2015).

### Chromatin Accessibility and Plant Hormone Signal

Epigenetic mechanisms have emerged as critical factors during SE (Osorio-Montalvo et al., 2018; Li et al., 2019; Wójcikowska et al., 2020). SE encounters DNA methylation variations by somatic cell reprogramming (De-la-Peña et al., 2015; Guo et al., 2020; Wang et al., 2020). 5-Azacytidine (5-AzaC), an inhibitor of DNA methylation, has been used during SE protocols (Osorio-Montalvo et al., 2018). The accumulation of chlorogenic acid (CGA), another kind of DNA methylation inhibitor, decreased −7.126 and − 5.264 (log_2_ FC) in IEC/CA and IOC/CA, respectively, indicating that chromatin accessibility and DNA methylation might play important roles in somatic cell differentiation (Osorio-Montalvo et al., 2018). Further, CGA correlated with DAPs in IEC/CA and IOC/CA remarkably (Supplementary Figure S9). The embryogenic nature of somatic cells is a prerequisite for auxin-induced chromatin accessibility and transcriptome alteration; auxin promotes the acquisition of plant cell totipotency in SE development pathway (Fraga et al., 2016; Wang et al., 2020; Wójcikowska et al., 2020). Plant growth regulators (PGRs) modify the levels of DNA methylation in the SE process, such as EC induction (De-la-Peña et al., 2015; Fraga et al., 2016). Auxin rapidly rewires the cell totipotency network by altering chromatin accessibility (Wang et al., 2020; Wójcikowska et al., 2020). Auxin-related PGRs have pleiotropic effects in cell proliferation and differentiation (Osorio-Montalvo et al., 2018; Ikeuchi et al., 2019; Wójcik et al., 2020). Endogenous active IAA plays an important role in the association between the expression of key factors inducing cell differentiation and the epigenetic regulatory network (Tang et al., 2020). PIC and 2,4-dichlorophenoxyacetic acid (2,4-D) are used for endogenous IAA biosynthesis and auxin signal regulation in plants, such as *A. praecox* and *A. thaliana* (Horstman et al., 2017). The inductive stimuli evoke endogenous auxin biosynthesis, leading to increased auxin levels and inducing totipotency in somatic cells (Su et al., 2021). Local auxin biosynthesis and polar auxin transport are essential for establishing auxin gradients during SE formation (Su et al., 2009, 2021; Bayer et al., 2017; Wójcik et al., 2020). It is widely accepted that the balance of auxin and cytokinin contents is critical for cell differentiation in different plants by regulating plant hormone signaling-related genes and methylation levels (Li et al., 2019; Zhao et al., 2021). However, few hormone-related DAMs were enriched in this study due to the extremely low hormone content in plant cells. Interestingly, the combination of PIC and 6-benzylaminopurine (6-BA) promoted organogenesis significantly (Supplementary Figure S10). The auxin gradients appear to activate PIN1 polar localization within the embryonic callus in *A*. *thaliana*, and polarized PIN1 is probably responsible for the polar auxin transport and accumulation in somatic embryos. The establishment of auxin gradients and PIN1-mediated polar auxin transport are essential for SE in *A*. *thaliana* (Su et al., 2009). The auxin- and CTK-related DEGs regulated SE capacity in plants, such as *G*. *hirsutum* (Xu et al., 2013) and *Passiflora edulis* (Rocha et al., 2015). In this study, auxin signal transduction-related DEGs including *auxin response factor* (*ARF*), *auxin/indoleacetic acids* (*AUX/IAAs*), *GH3*, and *transport inhibitor response1* (*TIR1*), CTK signal transduction-related genes (e.g., *ARR-A*, *ARR-B*, and *CRE1*) transcripts were differentially expressed with sucrose and maltose treatment (Supplementary Figure S11). Taken together, chromatin accessibility and DNA methylation, and plant hormone signal were critical for EC induction in *A. praecox*.

**Figure 8:**
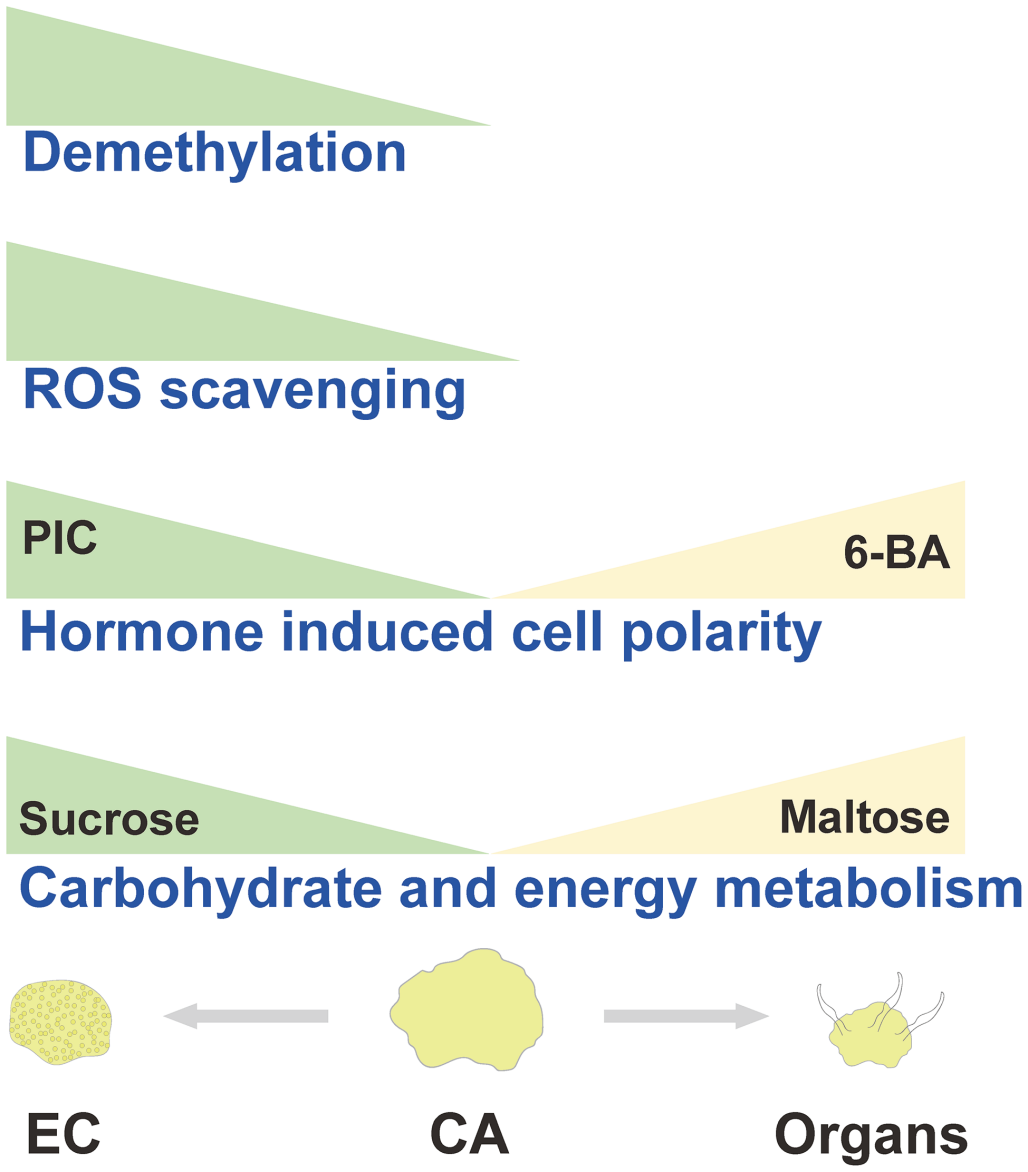
Hypothesized model diagram of callus differentiation direction in *A. praecox*.

Based on the aforementioned discussion, we hypothesized an EC induction model in *A. praecox* (Figure 8). In summary, CA undergone differentiation direction and stage showed different energy supply, stress response, hormone signal transduction, and chromatin accessibility level. All these differences might be responsible for the acquisition of embryogenic ability in *A. praecox*.

## Conclusion

Altogether, this study revealed valuable information regarding the molecular and biochemical changes that occurred during EC induction, and provided valuable foundation for comprehensive understanding of the mechanisms associated with SE and organogenesis in *A. praecox*. Using network analysis, we identified the potential key DAPs and DAMs with cell differentiation. Carbohydrate and energy metabolism was critical in controlling the transition between CA and EC, potentially by regulating basal metabolism, removal of ROS, chromatin accessibility, and plant hormonal signaling. We also assessed the roles of carbohydrate metabolism and plant hormone in cell differentiation direction. Thus, our results suggested that carbohydrate and energy metabolism cooperatively contributed to the activation cell totipotency and cell pluripotency, thereby inducing the EC and organs. Future research should focus on the possible interactions of carbohydrate metabolism, ROS responses and resistance, chromatin accessibility, and plant hormone signals, which might have a major impact on the acquisition of embryogenic ability in *A. praecox*.

## Data Availability Statement

The datasets presented in this study can be found in online repositories. The names of the repository/repositories and accession number(s) can be found at: National Center for Biotechnology Information (NCBI) BioProject database under accession number PRJNA800607. The proteomic data was deposited in ProteomeXchange (Dataset PXD031207).

## Author Contributions

YJ planned the study, analyzed the data, and wrote the manuscript. YD did sampling. YJ, YD, JY, and CL performed the experiments. SL, ZW, and SG revised the manuscript. All authors have read and agreed to the published version of the manuscript.

## Funding

This study was funded by the Science and Technology Innovation Team of Xinyang Agriculture and Forestry University (CXTD202002), the Horticultural Plant Biology Experimental Teaching Demonstration Center of Xinyang Agriculture and Forestry University (202102), and the Foundation of the Central Laboratory of Xinyang Agriculture and Forestry University (FCL202012).

## Conflict of Interest

The authors declare that the research was conducted in the absence of any commercial or financial relationships that could be construed as a potential conflict of interest.

## Publisher’s Note

All claims expressed in this article are solely those of the authors and do not necessarily represent those of their affiliated organizations, or those of the publisher, the editors and the reviewers. Any product that may be evaluated in this article, or claim that may be made by its manufacturer, is not guaranteed or endorsed by the publisher.

## Footnotes

^1^ http://www.kegg.jp/

^2^ https://www.oebiotech.com/

^3^ http://www.origin-gene.com/

## Abbreviations

SE: Somatic embryogenesis
EC: Embryogenic callus
SEs: Somatic embryos
non-EC: Non-embryogenic callus
DEGs: Differentially expressed genes
DAPs: Differentially accumulated proteins
DAMs: Differentially accumulated metabolites
CA: Callus
PIC: Picloram
IOC: Initial organogenesis-based callus
IEC: Initial embryogenic cell-originated callus
REC: Repetitive embryogenic cell-originated callus
GEC: Gradual embryogenic cell-originated callus
SEC: Subcultured-EC
BP: Bud primordium
ROS: Reactive oxygen species
APX: Ascorbate peroxidases
