# Supplementary material for: Integrated Proteomic and Metabolomic Analyses Provide Insights Into Acquisition of Embryogenic Ability in Agapanthus praecox
Source: Front Plant Sci. 2022 May 18;13:858065. doi: 10.3389/fpls.2022.858065 (PMC9158531; doi:10.3389/fpls.2022.858065)
Supplement: Supplementary file 8 [file Data_Sheet_1.pdf]

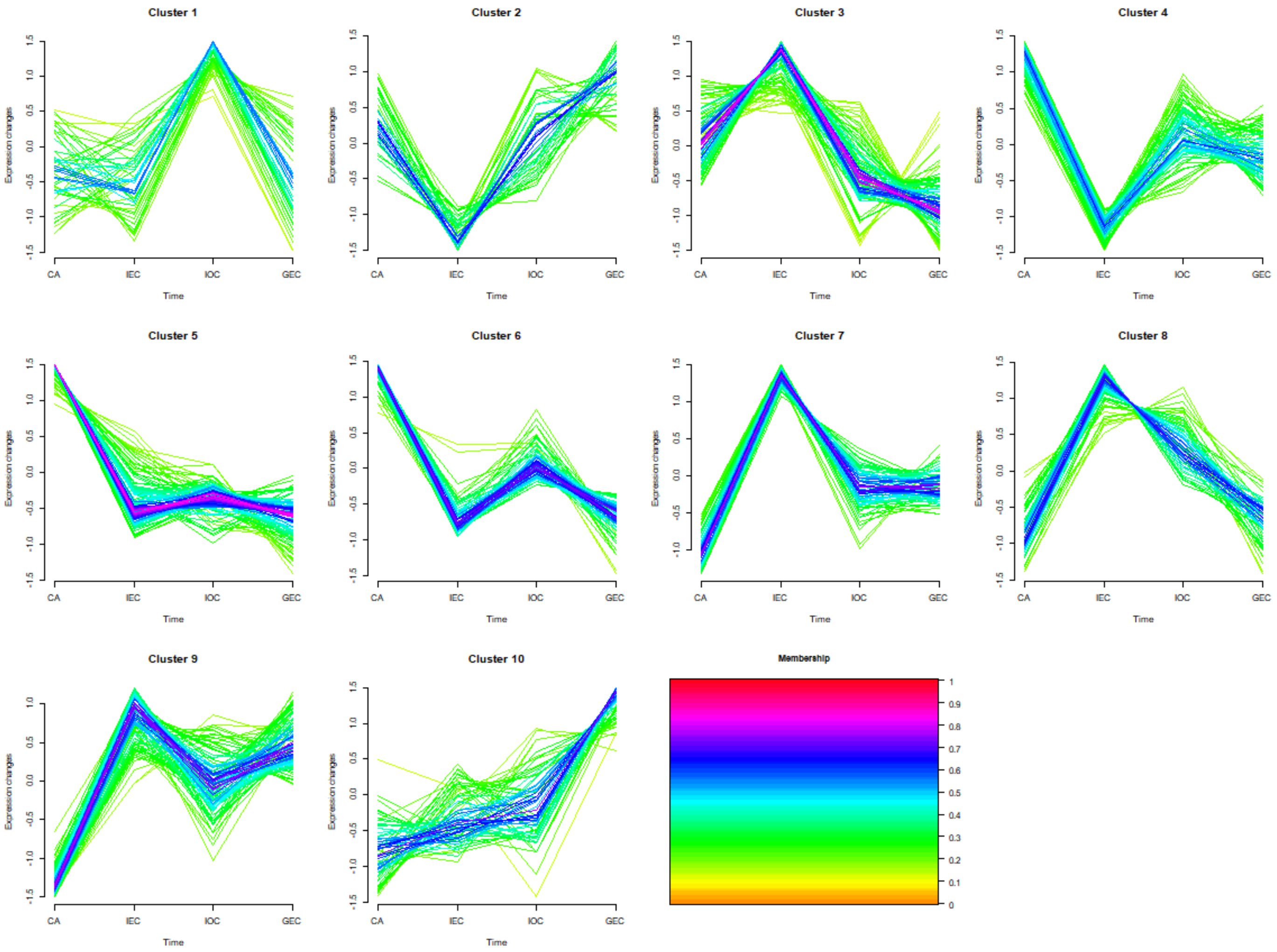

Supplementary Figure 1. Cluster analysis of differentially expressed proteins with different cell differentiation directions based on K-means method.

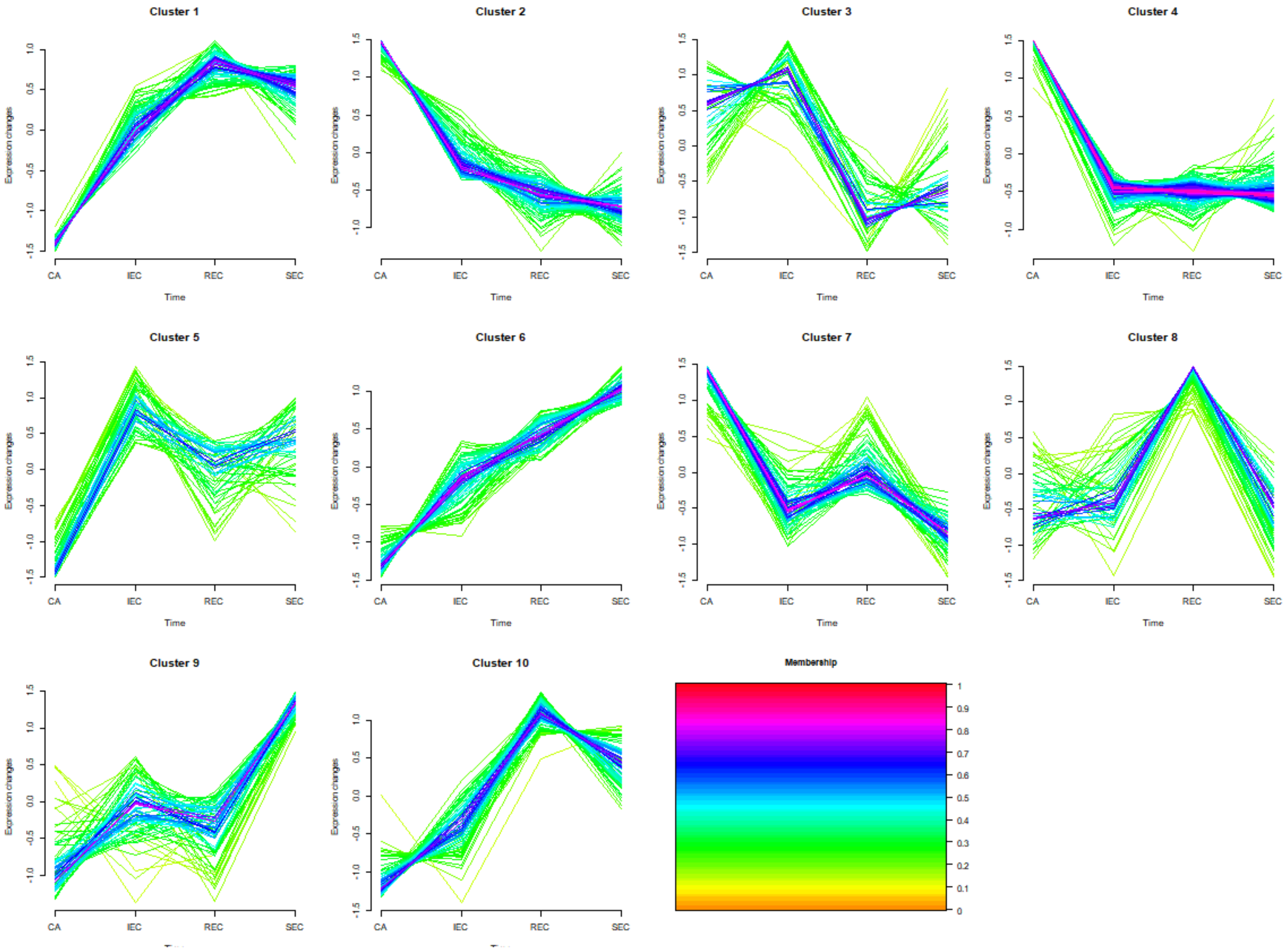

Supplementary Figure 2. Cluster analysis of differentially expressed proteins with different cell differentiation stages based on K-means method.

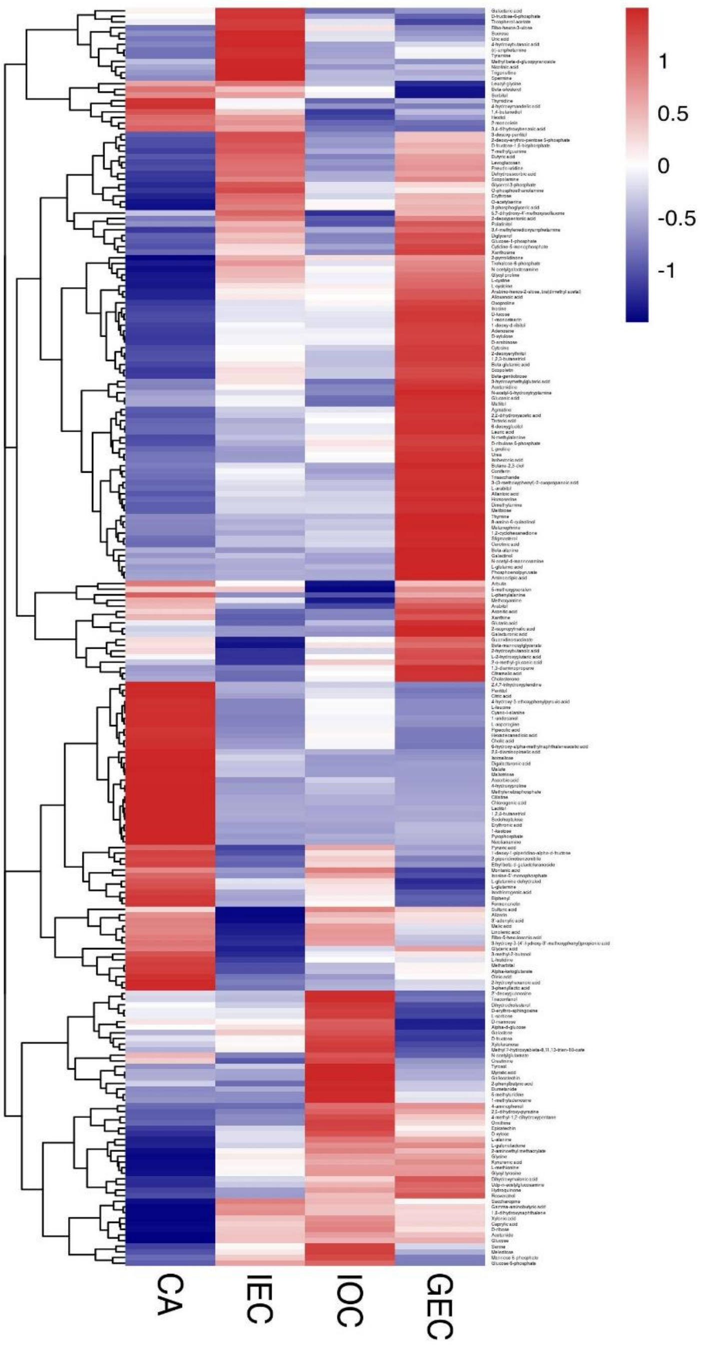

Supplementary Figure 3. Hierarchical clustering analysis and comparison of differentially accumulate metabolites in the directions of cell differentiation.

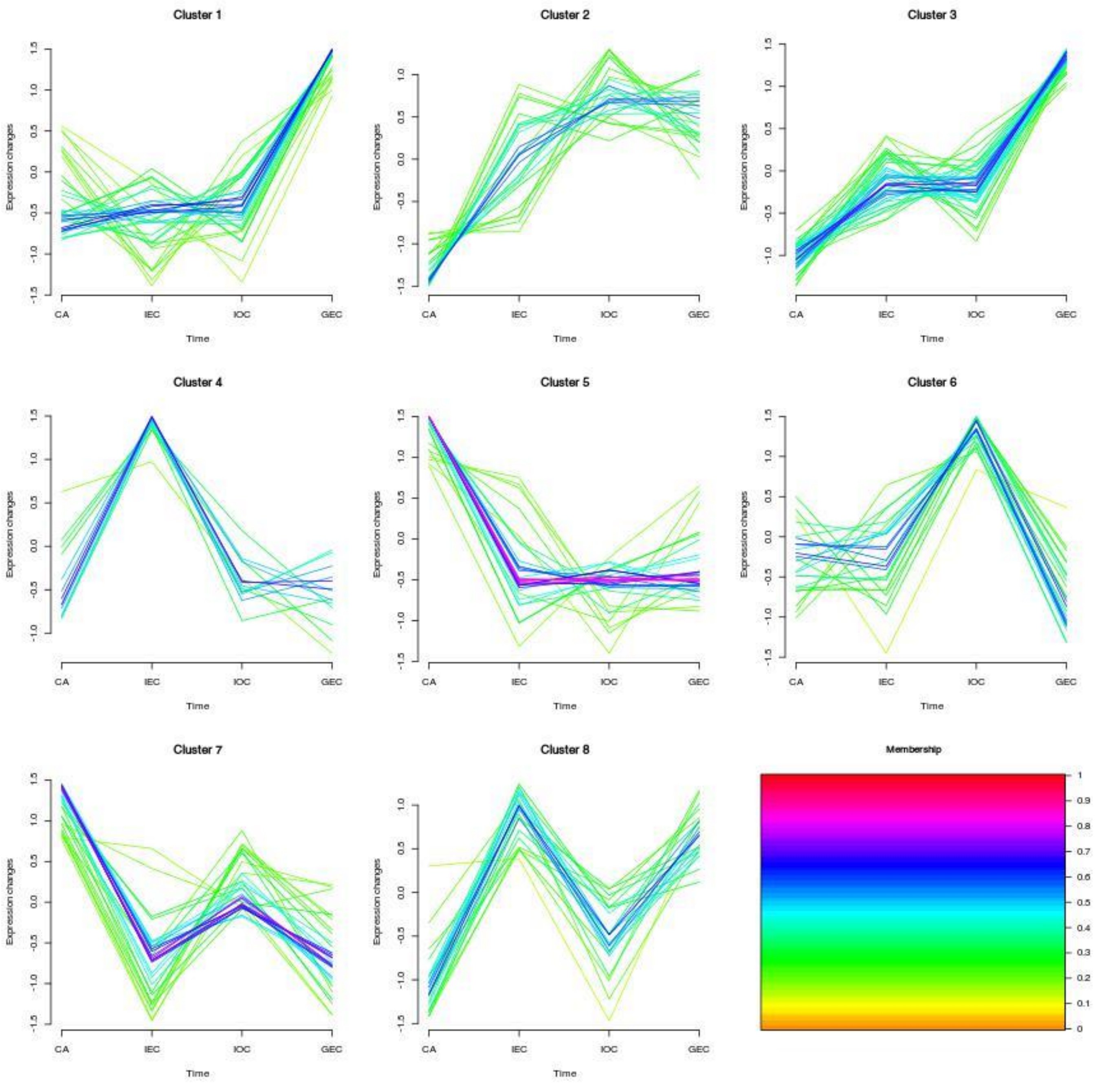

Supplementary Figure 4. Cluster analysis of differentially accumulate metabolites with different cell differentiation directions based on K-means method.

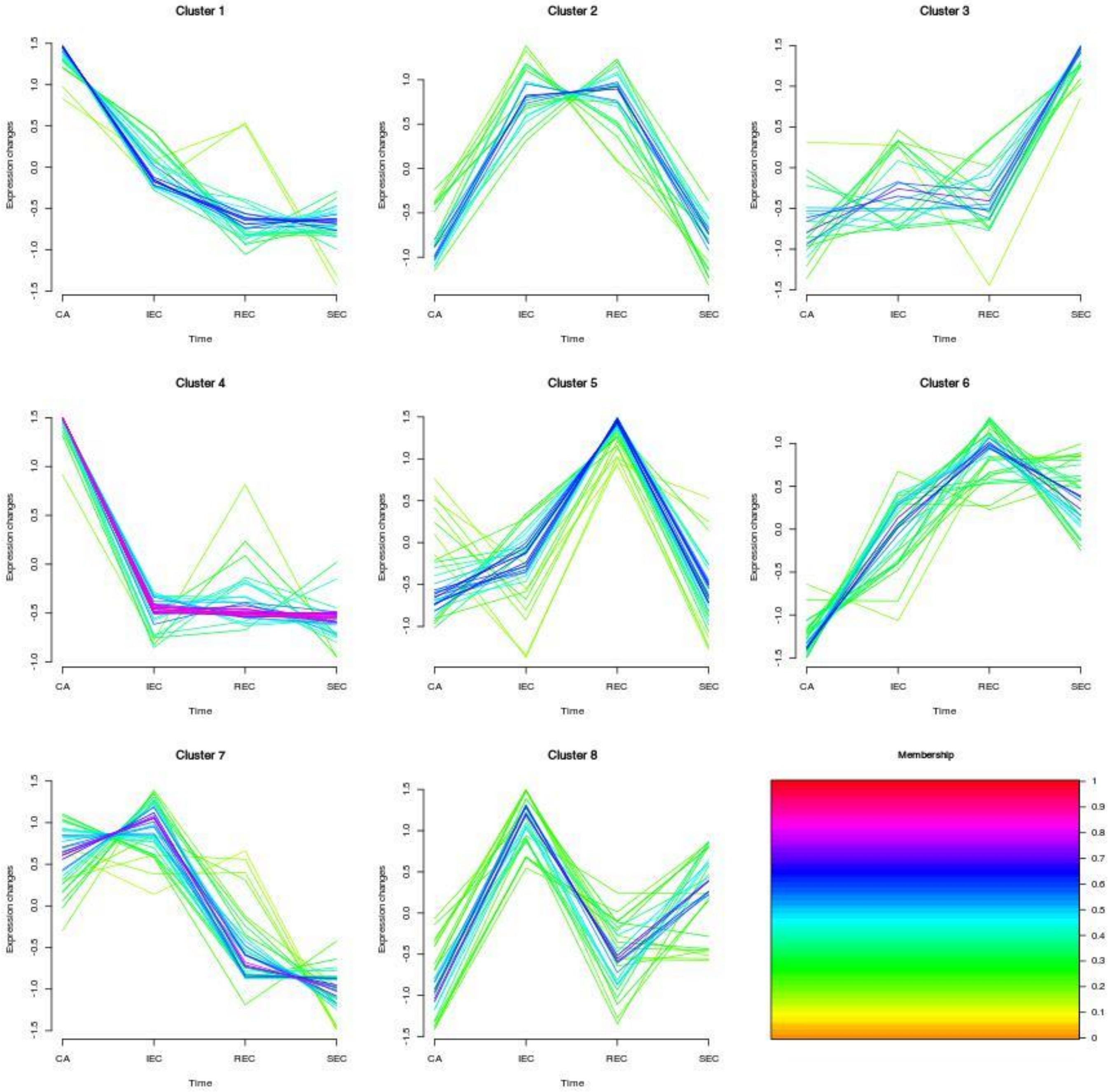

Supplementary Figure 5. Cluster analysis of differentially accumulate metabolites with different cell differentiation stages based on K-means method.

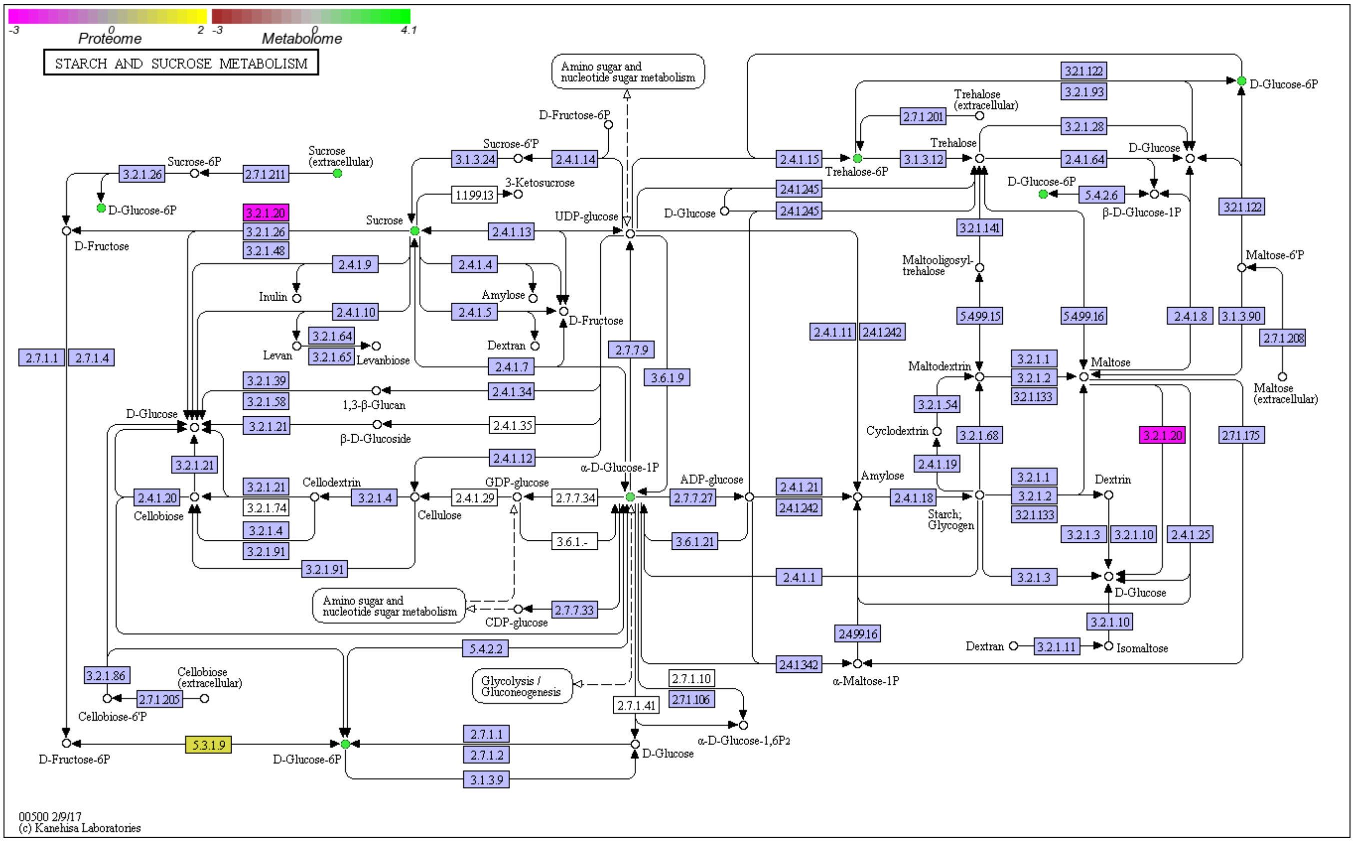

Supplementary Figure 6. Integrated proteomic and metabolomic analyses in the pathway of starch and sucrose metabolism.

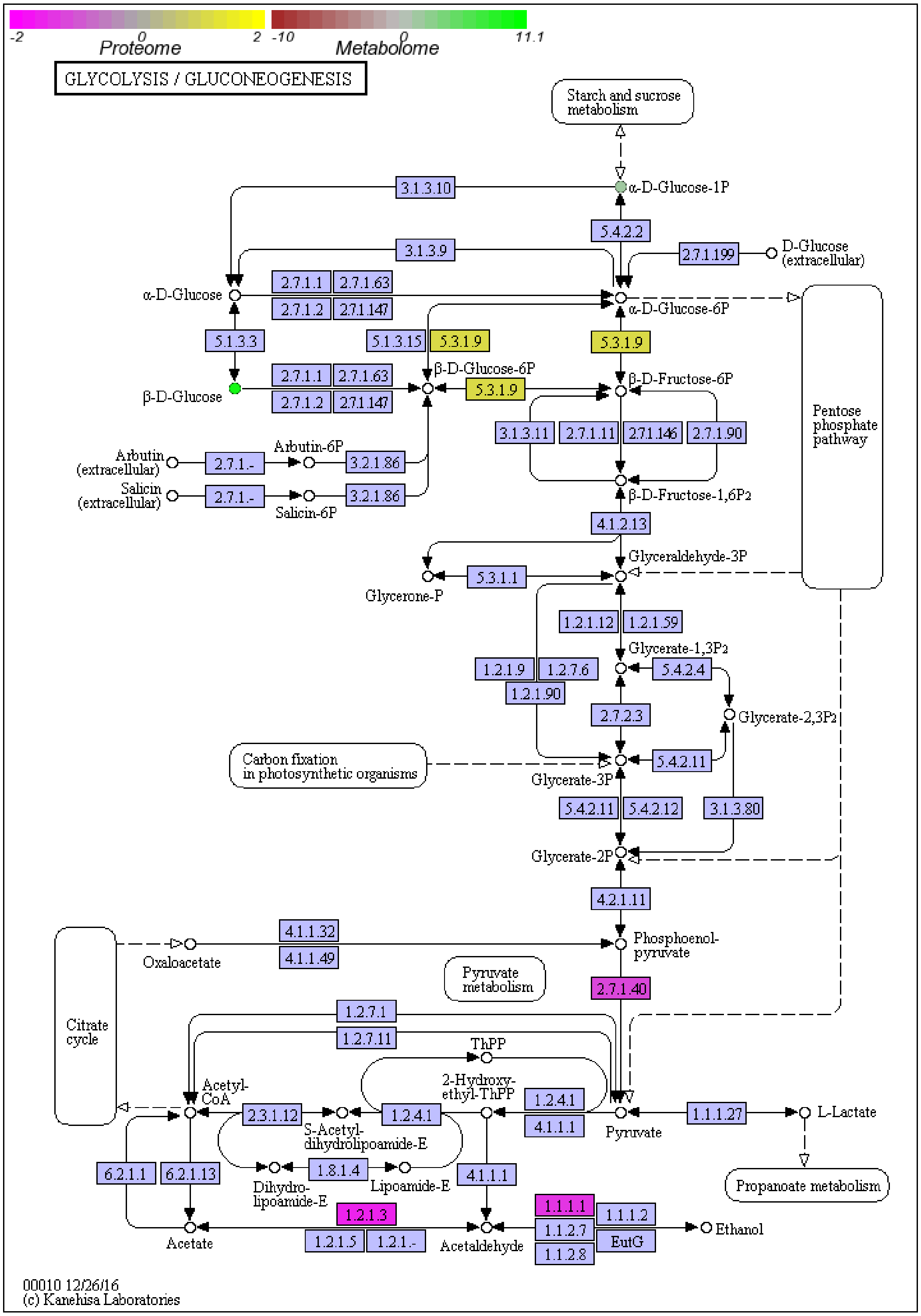

Supplementary Figure 7. Integrated proteomic and metabolomic analyses in the pathway of glycolysis/gluconeogenesis.

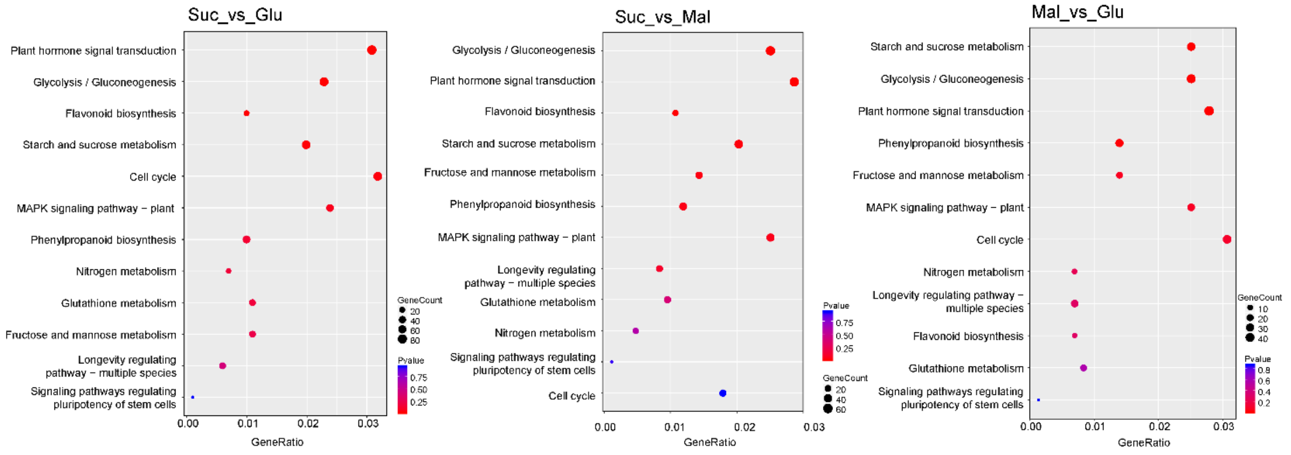

Supplementary Figure 8. KEGG pathway enrichment of the comparison of Suc/Glu, Suc/mal and Mal/Glu.

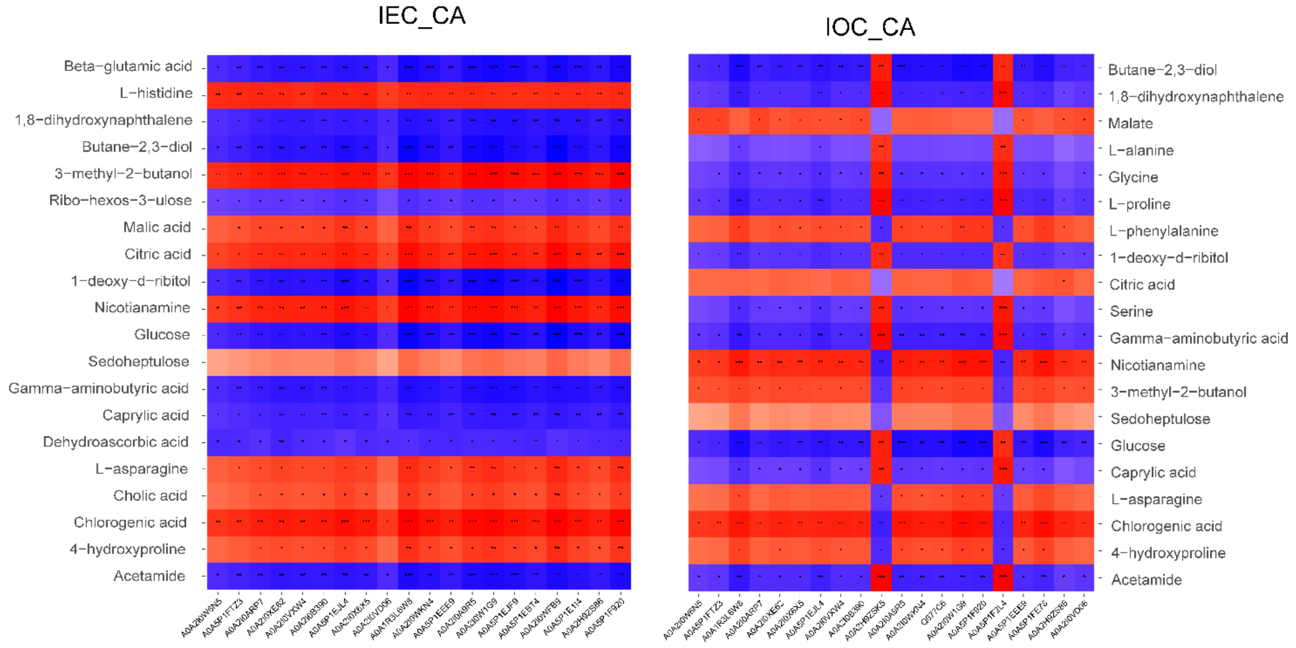

Supplementary Figure 9. Correlation analyses between the top 20 DEPs and DEMs with integrated proteomic and metabolomic analyses. The significance is shown at *P* < 0.05 (*), *P* < 0.01 (**) or *P* < 0.001 (***).

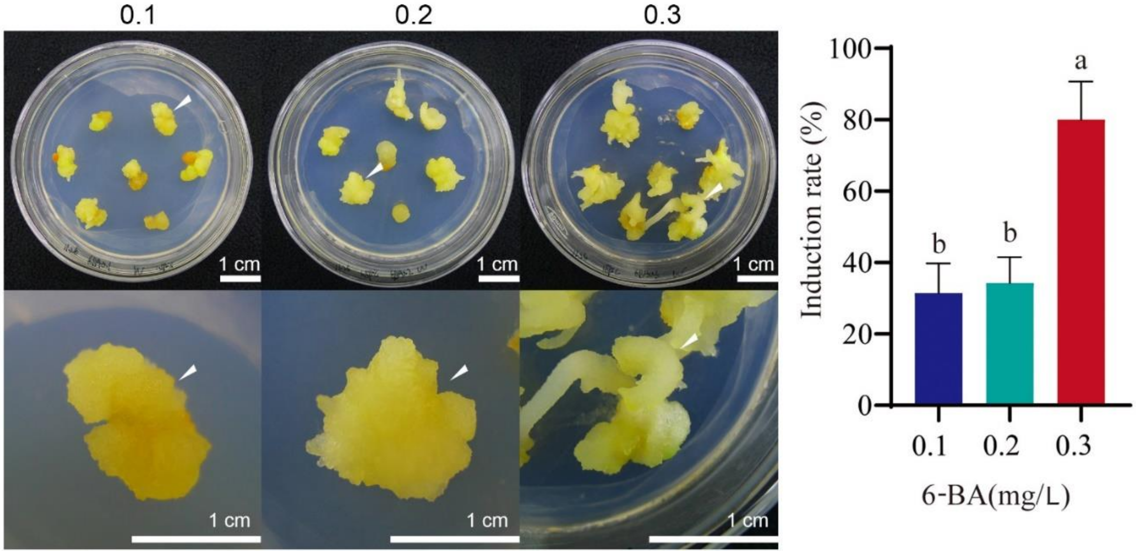

Supplementary Figure 10. Effects of the combination of picloram and 6-benzylaminopurine (6-BA) on cell differentiation direction. *n* = 3. Data on the bars marked without the same lowercase letter indicate significant differences at *P* < 0.05.

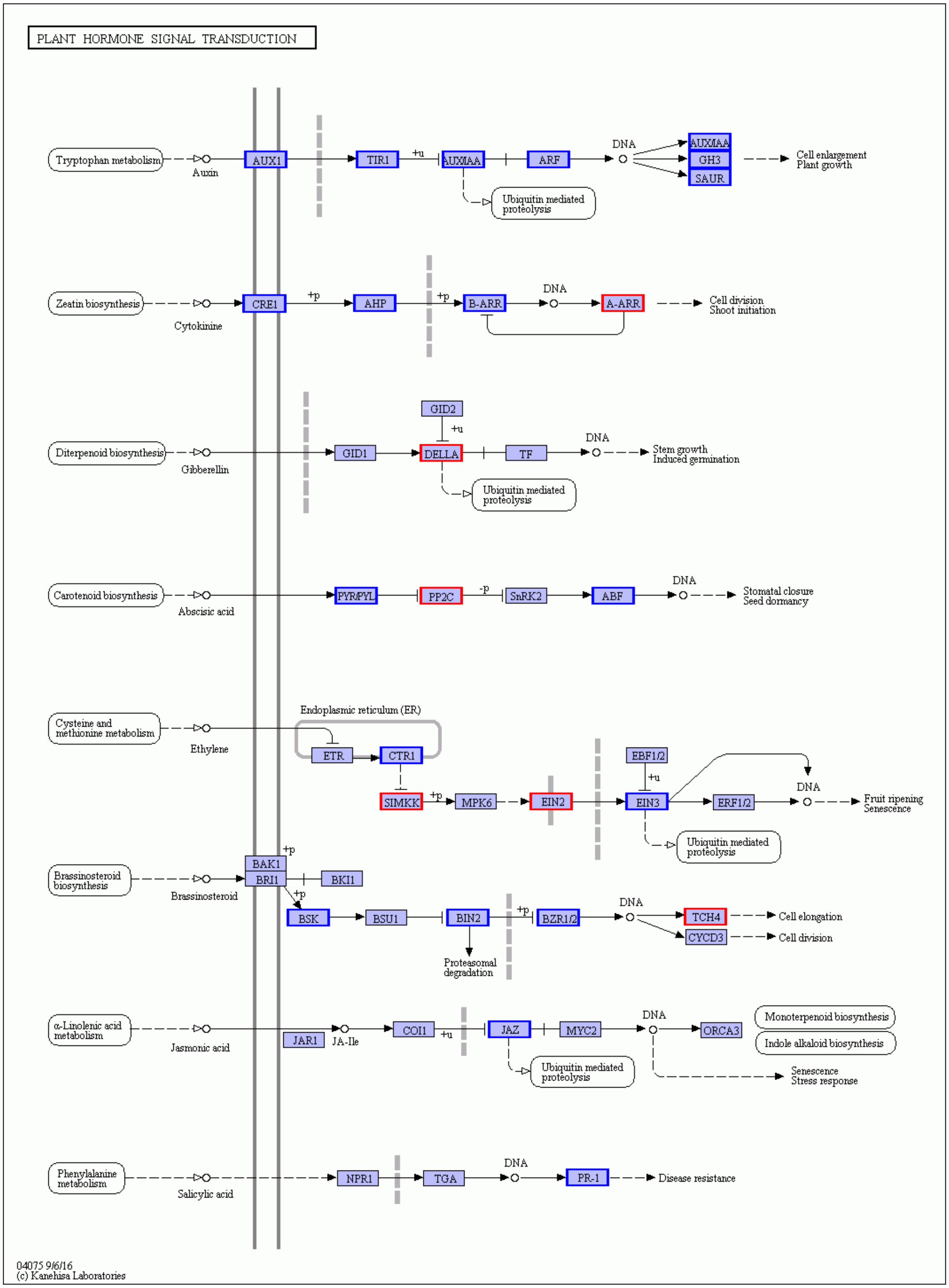

Supplementary Figure 11. Transcriptomic analyses between the sample of Suc and Mal in the pathway of plant hormone signal transduction.
